# The Use and Structure of Emergency Nurses’ Triage Narrative Data: Scoping Review

**DOI:** 10.2196/41331

**Published:** 2023-01-13

**Authors:** Christopher Picard, Manal Kleib, Colleen Norris, Hannah M O'Rourke, Carmel Montgomery, Matthew Douma

**Affiliations:** 1 Faculty of Nursing University of Alberta Edmonton, AB Canada; 2 School of Nursing Midwifery and Health Systems University College Dublin Dublin Ireland

**Keywords:** nursing, artificial intelligence, machine learning, triage, review, narrative

## Abstract

**Background:**

Emergency departments use triage to ensure that patients with the highest level of acuity receive care quickly and safely. Triage is typically a nursing process that is documented as structured and unstructured (free text) data. Free-text triage narratives have been studied for specific conditions but never reviewed in a comprehensive manner.

**Objective:**

The objective of this paper was to identify and map the academic literature that examines triage narratives. The paper described the types of research conducted, identified gaps in the research, and determined where additional review may be warranted.

**Methods:**

We conducted a scoping review of unstructured triage narratives. We mapped the literature, described the use of triage narrative data, examined the information available on the form and structure of narratives, highlighted similarities among publications, and identified opportunities for future research.

**Results:**

We screened 18,074 studies published between 1990 and 2022 in CINAHL, MEDLINE, Embase, Cochrane, and ProQuest Central. We identified 0.53% (96/18,074) of studies that directly examined the use of triage nurses’ narratives. More than 12 million visits were made to 2438 emergency departments included in the review. In total, 82% (79/96) of these studies were conducted in the United States (43/96, 45%), Australia (31/96, 32%), or Canada (5/96, 5%). Triage narratives were used for research and case identification, as input variables for predictive modeling, and for quality improvement. Overall, 31% (30/96) of the studies offered a description of the triage narrative, including a list of the keywords used (27/96, 28%) or more fulsome descriptions (such as word counts, character counts, abbreviation, etc; 7/96, 7%). We found limited use of reporting guidelines (8/96, 8%).

**Conclusions:**

The breadth of the identified studies suggests that there is widespread routine collection and research use of triage narrative data. Despite the use of triage narratives as a source of data in studies, the narratives and nurses who generate them are poorly described in the literature, and data reporting is inconsistent. Additional research is needed to describe the structure of triage narratives, determine the best use of triage narratives, and improve the consistent use of triage-specific data reporting guidelines.

**International Registered Report Identifier (IRRID):**

RR2-10.1136/bmjopen-2021-055132

## Introduction

### Overview

There are an estimated 46.6 emergency department (ED) visits per 100 people in the United States or 142 million annual visits to Canadian and American EDs combined [1,2]. EDs sort and prioritize patients using triage to ensure that patients with the highest level of acuity are provided care quickly and safely. Modern electronic health records allow for the large-scale collection of triage data, such as time stamps, vital signs, screening assessments, and free-text descriptions [3,4]. These data can be used to track ED volumes and guide local and national policy decisions [5]. Machine learning (ML) and artificial intelligence have allowed the data to be examined for a range of purposes [5,6]. Despite the ubiquity of triage and triage-related data collection, the potential research impact of using triage narrative data remains largely unrealized [7,8].

### Background

Triage is the process of sorting patients. It originated during the Napoleonic wars [9] and was introduced into civilian practice in the 1960s [10]. Triage was formalized using validated tools in the 1980s [11] and was first implemented in Australia as a national system in 1994 [12]. Most countries use a formal triage system [13] associated with improved patient safety and service efficiency outcomes [14]. Triage is typically performed by experienced ED nurses [15] who are specially trained to use formally validated triage assessment tools to prioritize patient care [13]. Triage assessment typically consists of a brief history and physical assessment of the patient, followed by the assignment of a visit category and triage priority level by the nurse [15].

Several countries have standardized the mandatory collection of ED data. Canadian [16,17] and Australian [18] EDs report a triage minimum data set of structured complaint code fields. In addition to these nationally coordinated triage data collection efforts, there are regional databases for the local monitoring of injuries or syndromic surveillance (eg, toxic drug supplies and infectious disease outbreaks) [19]. The triage data collected between systems will vary, but the data types can be grouped into either structured or unstructured data, with each data type having its own strengths and weaknesses.

Structured data force the triage nurse to select from one of several preformatted options and restrict the types of data that can be entered into any given data field. Examples of structured triage data include arrival time, vital signs, demographic information (ie, age and sex), insurance status, categorical chief complaints, and numerical triage acuity score. Structured data are the most frequently reported data generated during triage [4,5]. Structured data are readily available (owing to their routine collection) and simple to analyze and report compared with unstructured data; however, this convenience comes at a loss of contextual detail that is available from unstructured narratives [5].

Unstructured clinical data include free-text written notes or “narrative” [20]. Narratives generated at triage vary in length and structure depending on the electronic health record and triage system used. The narrative typically includes the triage nurse’s assessment and the patient’s reported reason for visiting the ED. These data are unstructured and allow nurses to record the chief complaint in the patient’s own words, descriptions of events associated with the ED presentation, and their physical examination findings [21].

Two systematic reviews that focused on injuries examined whether unstructured clinical narratives, including those generated at triage, could be used for large-scale injury surveillance [22,23]. These reviews summarized how narrative data were used to gather injury information and highlighted how data fields were interrogated [22,23]. Cumulatively, the reviews examined 2831 studies published over 18 years and included 56 studies, 13 of which used ED triage data [22,23]. They reported that narrative data use has increased over time and that analyzing the data required automatic or manual extraction of keywords or ML techniques. The review authors were critical of data heterogeneity and called for improved data collection methods [22,23]. The heterogeneity noted in these studies may be partially explained by the wide range of administrative data set types interrogated. A more homogeneous data set (ie, triage narratives alone) may have offered alternative insights.

Two additional review studies published in 2013 focused their analyses on studies using triage narratives for syndromic surveillance systems (ie, programs that monitor for disease outbreaks) [19,24]. Syndromic classifiers use chief complaint narratives to group patient visits into categories to monitor for changes (eg, outbreaks) in disease presentations. The first systematic review screened 89 studies identified through a structured search limited to PubMed to examine syndromic classifiers for detecting influenza in ED triage data sets [24]. The authors included 38 studies that met their inclusion criteria: (1) examined clinical data, which was (2) generated in the ED, and (3) examined influenza. The most commonly used method to identify cases was chief complaint classification. The authors noted that ED triage narratives allowed for large-scale research and program evaluation, but no details on the structure of or methods for extracting chief complaint classification data were offered [24].

The second 2013 nonsystematic review also focused on syndromic surveillance. This review offered descriptive details on the structure of syndromic surveillance systems and their data [19]. The review included 17 studies drawn from an undisclosed initial sample and identified 15 chief complaint classifier systems of interest. The authors described the geographic location where each system was in use and the process used by each system to group visits into syndromes and detailed the relative strengths and weaknesses of each system. The review noted that all but 1 system (from Canada) was American and that the classifiers used differing degrees of computer text parsing to assign patients into groups (eg, ranged from 4 to 9 syndromes) and classified the approach of each system by keyword, statistical, or linguistic methods. The authors highlighted that statistical methods relied on large data volumes to be robust to the “noisy” inputs found in narrative text. By contrast, keyword and linguistic methods used keyword-based strategies and were described as disadvantageous because time-intensive adjustments were needed to accommodate variations in triage vocabulary. The drawbacks of keyword-based methods were balanced by the transparency offered when compared with ML studies. The authors argued that triage narratives are of great utility for disease surveillance and were less critical of variations in the initial data quality, concluding that there is a need for common syndromic definitions to improve the utility of these data.

Despite the use of triage data for multiple purposes, there is a criticism of the methods used to classify triage narratives and a call for improved consistency and quality in their collection. There are documented efforts to create common data definitions for triage narratives [25] and to create national ED nursing data sets [26]; however, unstructured data are not as widely collected as structured data [7], and there is a paucity of literature examining what structures are common to triage narratives. This scoping review addresses these concerns and examines peer-reviewed literature to identify what ED triage narrative data have been used for, studies that may be sufficiently similar to compare, and the need for additional research. This scoping review systematically examines the evidence to determine what, if any, structures underlie these narrative data and describes what the data have been used for.

### Objectives

The objectives of this review were as follows:

1. Describe the current literature on the use of ED nurses’ triage narratives
2. Describe the objectives and findings of the included studies
3. Determine whether there are sufficient data to systematically review the structure or descriptions of triage narratives
4. Determine whether there is adequate consistency in the included studies to support further review of the outcomes.

## Methods

### Overview

In this review, we used the scoping framework proposed by Arksey and O’Malley [27,28]. The protocol was published previously [29]. The PRISMA-ScR (Preferred Reporting Items for Systematic Reviews and Meta-Analyses Extension for Scoping Reviews) framework was used to guide reporting [30]. To identify studies that examined unstructured narratives in the ED, we conducted a search using controlled terminology for the main topics of health record narratives, emergency, and triage. A medical librarian refined the search terms, and prespecified filters were used for ED [31-34]. To maximize the breadth of the retrieved studies, a comprehensive search was conducted in CINAHL, Ovid MEDLINE, Ovid Embase, Cochrane Library (via Wiley), and ProQuest Central. The search was limited to peer-reviewed literature published after 1990, four years before the first nationally implemented triage system [12]. The reference lists of select excluded studies, namely those that examined the free-text narratives of emergency physicians and review studies that included triage narratives, were hand searched for inclusion. There were no deviations from the published protocol [29].

Data were downloaded into Covidence (Veritas Health Innovation) for screening. The studies were screened independently by 2 authors (CP and MJD) in 2 stages (title plus abstract and then full text) using prepiloted screening forms. Any peer-reviewed studies that examined unstructured narratives [22,35] that were generated within an ED [36] by a nurse [37] were included. Studies that examined disaster triage systems, studies that did not have full text (ie, abstracts only), and non-English studies were excluded. Cohen κ was used to gauge agreement during screening, and all conflicts were settled by consensus. There were no deviations from the study protocol, which outlined the screening forms and operational definitions [29].

### Data Extraction

The data were extracted into Microsoft Excel (version 2019, Microsoft Corp; by CP) using prepiloted forms. The results were independently confirmed by a second reviewer (MJD). Counts and proportions were used to describe categorical and numeric values. The extracted categorical values included study variables such as study design, country of origin, triage system used, and the use of ML. The extracted numerical data included the publication year, number of EDs from which the data were drawn, number of visits or patients included in the initial and final samples, and the number of nurses included in each study. For studies that reported data as minimum values (ie, “there were over three million of visits”) [38-45], values were recorded as the minimum stated value (ie, 3 million). When studies reported using quality or reporting frameworks, we reported the tool by name. The main conceptual categories of each study (ie, the objectives, design, population, and results) were described [46]. We summarized the descriptions of the triage narratives and keywords when the narratives were reported in the study. When 5 or fewer keywords were used, they were recorded verbatim.

### Data Analysis

Owing to the wide distribution of data, estimates of central measures were calculated using both median (with IQRs) and minimum and maximum counts. Statistical analyses were performed using SPSS (version 25, IBM Corp). Citation management was performed using Zotero (Corporation for Digital Scholarship). The study objectives were categorized dichotomously (ie, yes or no) based on whether ML was used in the study (defined as any form of artificial intelligence), and the y were grouped into exclusive categories according to the primary use of the triage narratives: case identification, predictor variable, or quality improvement.

## Results

### Overview of Studies

**Figure 1:**
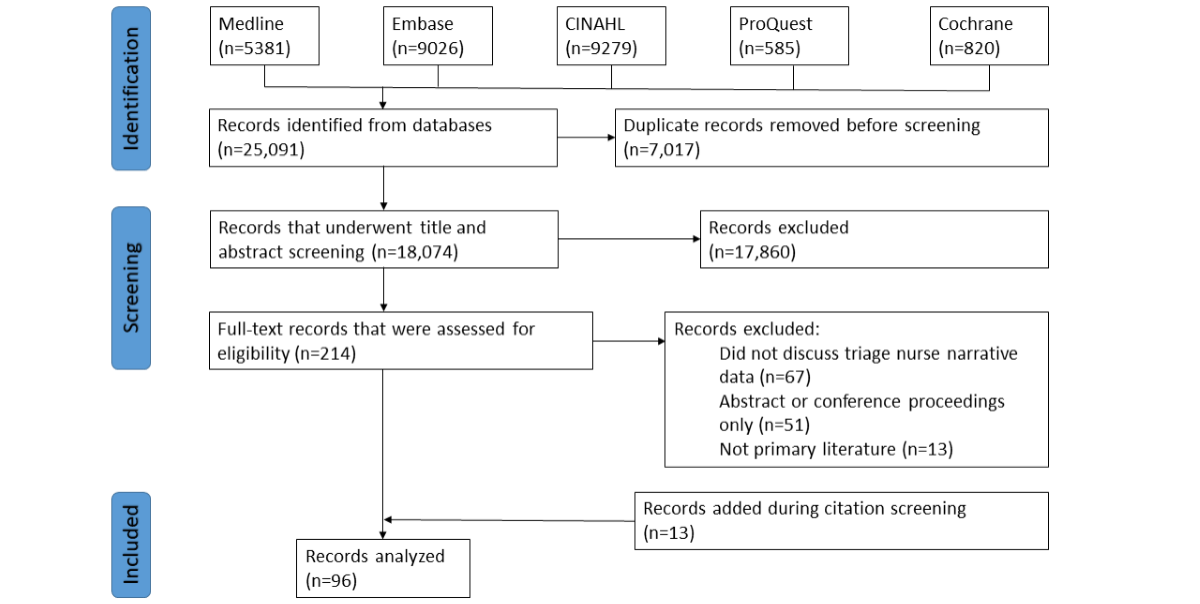
PRISMA diagram.

A total of 25,091 studies were identified in the initial search, and after deduplication, 18,074 (72.03%) studies underwent title and abstract screening. The proportionate agreement between reviewers (CP and MJD) during screening was 97.4% for the excluded studies and 98.1% for the included studies (Cohen κ=0.250). A full-text review was completed for 214 studies, and 131 (61.2%) studies were excluded at this stage, primarily for not specifying whether the narratives were generated by a nurse at triage (67/131, 51.1%). All review studies identified in the initial search that discussed narrative (although excluded) underwent citation screening in the primary search that discussed triage or ED narratives underwent citation screening. An additional 13 studies were included at this stage (Figure 1).

### Study Designs

Retrospective design was the most common approach (80/96, 83%; Multimedia Appendix 1). Data were typically drawn (in part or entirely) from electronic databases, except in earlier studies, in which data were manually abstracted from paper charts [47-49]. The studies used data from hospitals (63/96, 66%) or regional databases (33/96, 34%). All studies reported on the unstructured narratives generated at triage; however, there was significant variation in the types and details of additional data reported. The most commonly collected non–triage-narrative data were patient demographic data, namely age (63/96, 66%), sex (60/96, 62%), and vital signs (29/96, 30%); visit details, namely chief complaint codes (57/96, 59%), discharge status (53/96, 55%), arrival date (35/96, 36%), and time (32/96, 33%); and ED data, namely triage system used (41/96, 43%; Multimedia Appendix 2 [22,36]). There was a weak relationship between the number of items collected and time, with 12% (*R*^2^=0.122) of the variation being attributable to publication date (*r*_94_=0.35; *P*<.001). The number of EDs included was reported in 92% (88/96) of studies. The initial data set size was reported in 81% (78/96) of studies. Of the 96 included cases, 76 (79%) reported the number of visits, and 28 (29%) reported the number of patients. The number of nurses who generated the narratives used in the study was reported in 9% (9/96) of studies [38,48-55].

**Figure 2:**
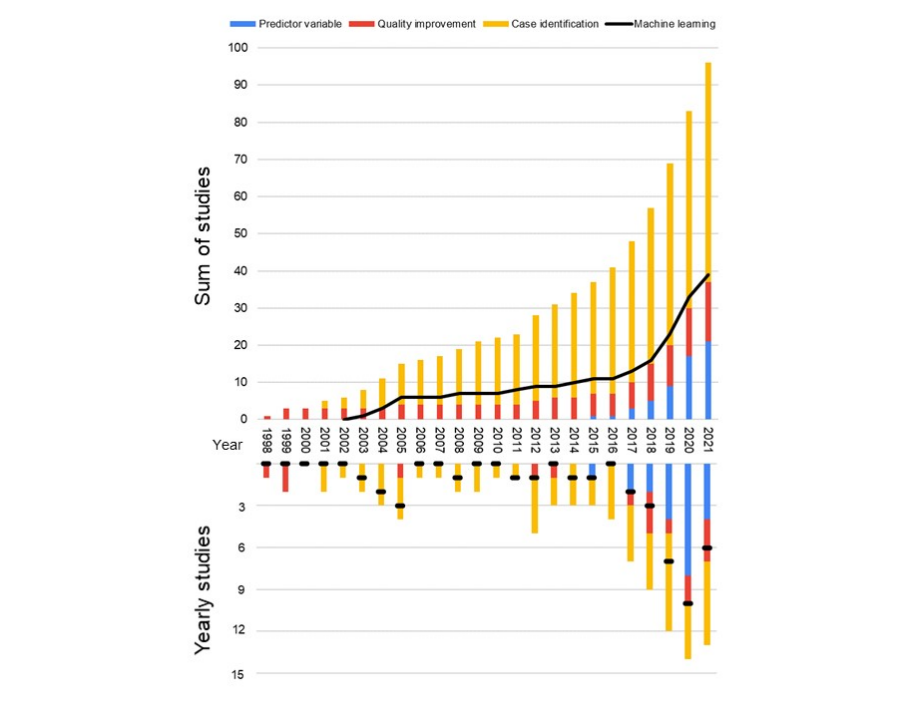
Triage narrative uses.

The median study size included 12,103 (IQR 803-150,089) visits or 391 (IQR 391-76,069) patients from an initial sample of 60,231 (IQR 2943-461,435) visits from (IQR 1-12) 2 EDs (Table 1). There was a large variation in the numbers of visits and departments examined, with the included sample sizes ranging from fewer than 100 to >2 million visits. These visits were drawn from databases ranging from 100 to >14 million visits and reflected as few as 1 ED and as many as 496 EDs (Table 1). There was an increase in the number of studies performed and median sample size of studies in each 6-year period between 1998 and 2021, with 61% (59/96) of the studies published in the last 6 years, that is, after 2015. The median sample sizes increased after 2009 from 7951 (IQR 518-55,952) to 160,944 (IQR 19,418-501,758). There was a concurrent increase in the frequency of ML use as a primary tool, with 77% (30/39) of studies after 2017 using ML use as a primary tool (Table 1). We noted that ML was used more frequently in predictive studies (21/25, 84%) than in studies using narratives for case identification (17/58, 29%) or quality (1/13, 8%; Figure 2).

Geographically, the United States (43/96, 45%), Australia (31/96, 32%), and Canada (5/96, 5%) represented 82% (79/96) of the published papers; 1 study was reported each from South America and Africa (Table 2). The studies coming from countries with official languages other than English [51,54,56-59] were from countries that adopted or adapted the existing triage systems. Other countries with large English-speaking populations are either underrepresented (England and New Zealand) or not represented at all (South Africa, Wales, Ireland, and Scotland; Multimedia Appendix 1).

**Table 1:** Study characteristics by publication year.

		Studies (n=96)	Included EDs^a^			Initial sample			Included visits			Included patients			Included nurses			Studies using ML^b^ methods (n=39)
			Total^c^ (n=2438)	Studies^d^ (n=88)	Total^c^ (n=63,413,919)		Studies^d^ (n=78)	Total^c^ (n=12,220,866)		Studies^d^ (n=76)	Total^c^ (n=1,804,813)		Studies^d^ (n=28)	Total^c^ (n=3844)		Studies^d^ (n=9)		
**Year, n (%)**																		
	1998	1 (1.04)	1 (0.04)	1 (1.14)	104 (0.0001)		1 (1.28)	104 (0.0008)		1 (1.32)	104 (0.01)		1 (3.57)	0 (0)		0 (0)	0 (0)	
	1999	2 (2.08)	2 (0.08)	2 (2.27)	100 (0.001)		2 (2.56)	100 (0.0008)		2 (2.63)	100 (0.01)		2 (7.14)	24 (0.62)		2 (2.22)	0 (0)	
	2000	0 (0)	0 (0)	0 (0)	0 (0)		0 (0)	0 (0)		0 (0)	0 (0)		0 (0)	0 (0)		0 (0)	0 (0)	
	2001	2 (2.08)	497 (20.39)	2 (2.27)	98,672 (0.16)		2 (2.56)	84,000 (0.69)		1 (1.32)	0 (0)		0 (0)	0 (0)		0 (0)	0 (0)	
	2002	1 (1.04)	1 (0.04)	1 (1.14)	11,861 (0.02)		1 (1.28)	0 (0)		0 (0)	305 (0.02)		1 (3.57)	0 (0)		0 (0)	0 (0)	
	2003	2 (2.08)	5 (0.21)	2 (2.27)	43,078 (0.07)		1 (1.28)	17,413 (0.14)		2 (2.63)	0 (0)		0 (0)	0 (0)		0 (0)	1 (2.56)	
	2004	3 (3.12)	23 (0.94)	3 (3.41)	1,021,949 (1.61)		3 (3.85)	21,949 (0.18)		2 (2.63)	73,115 (4.05)		2 (7.14)	0 (0)		0 (0)	2 (5.13)	
	2005	4 (4.17)	14 (0.57)	3 (3.41)	579,032 (0.91)		3 (3.85)	1510 (0.01)		2 (2.63)	86,079 (4.77)		2 (7.14)	0 (0)		0 (0)	3 (7.69)	
	2006	1 (1.04)	1 (0.04)	1 (1.14)	46,602 (0.07)		1 (1.28)	45,329 (0.37)		1 (1.32)	0 (0)		0 (0)	50 (1.3)		1 (11.11)	0 (0)	
	2007	1 (1.04)	1 (0.04)	1 (1.14)	521 (0.0008)		1 (1.28)	419 (0.003)		1 (1.32)	0 (0)		0 (0)	0 (0)		0 (0)	0 (0)	
	2008	2 (2.08)	95 (3.9)	2 (2.27)	119,479 (0.19)		2 (2.56)	5956 (0.05)		2 (2.63)	0 (0)		0 (0)	0 (0)		0 (0)	1 (2.56)	
	2009	2 (2.08)	14 (0.57)	2 (2.27)	3,556,352 (5.61)		2 (2.56)	1,089,984 (8.92)		1 (1.32)	389 (0.02)		1 (3.57)	0 (0)		0 (0)	0 (0)	
	2010	1 (1.04)	2 (0.08)	1 (1.14)	263,937 (0.42)		1 (1.28)	19,252 (0.16)		1 (1.32)	0 (0)		0 (0)	0 (0)		0 (0)	0 (0)	
	2011	1 (1.04)	6 (0.25)	1 (1.14)	794 (0.001)		1 (1.28)	794 (0.001)		1 (1.32)	0 (0)		0 (0)	2 (0.05)		1 (11.11)	1 (2.56)	
	2012	5 (5.21)	182 (7.47)	5 (5.68)	12,810,122 (20.2)		3 (3.85)	71,427 (0.58)		4 (5.26)	519 (0.03)		1 (3.57)	27 (0.7)		2 (22.22)	1 (2.56)	
	2013	3 (3.12)	4 (0.16)	2 (2.27)	348,895 (0.55)		1 (1.28)	41,624 (0.34)		1 (1.32)	798 (0.04)		1 (3.57)	0 (0)		0 (0)	0 (0)	
	2014	3 (3.12)	282 (11.57)	3 (3.41)	16,074,953 (25.35)		3 (3.85)	43,114 (0.35)		2 (2.63)	38,479 (2.13)		1 (3.57)	3538 (92.04)		1 (11.11)	1 (2.56)	
	2015	3 (3.12)	74 (3.04)	3 (3.41)	13,051,141 (20.58)		2 (2.56)	310,353 (2.54)		3 (3.95)	0 (0)		0 (0)	0 (0)		0 (0)	1 (2.56)	
	2016	4 (4.17)	109 (4.47)	3 (3.41)	13,194 (0.02)		3 (3.85)	2972 (0.02)		2 (2.63)	369 (0.02)		1 (3.57)	0 (0)		0 (0)	0 (0)	
	2017	7 (7.29)	345 (14.15)	5 (5.68)	2,450,310 (3.86)		5 (6.41)	2,287,592 (18.72)		7 (9.21)	0 (0)		0 (0)	0 (0)		0 (0)	2 (5.13)	
	2018	9 (9.38)	18 (0.74)	8 (9.09)	195,014 (0.31)		8 (10.26)	59,801 (0.49)		8 (10.53)	183 (0.01)		2 (7.14)	10 (0.26)		1 (11.11)	3 (7.69)	
	2019	12 (12)	641 (26.29)	10 (11.36)	5,453,665 (8.6)		10 (12.82)	3,426,182 (28.04)		10 (13.16)	153,145 (8.49)		3 (10.71)	193 (5.02)		1 (11.11)	7 (17.95)	
	2020	14 (14.58)	29 (1.19)	14 (15.91)	4,183,453 (6.6)		12 (15.38)	3,372,239 (27.6)		10 (13.16)	1,029,147 (57.02)		7 (25)	0 (0)		0 (0)	10 (25.64)	
	2021	13 (13.54)	92 (3.77)	13 (14.77)	3,090,691 (4.87)		10 (12.82)	1,318,752 (10.79)		12 (15.79)	422,081 (23.39)		3 (10.71)	0 (0)		0 (0)	6 (15.38)	
Value, median (IQR)^e^		2.5 (1-4.25)	2 (1-12)	N/A^f^	60,231 (2943-461,435)		N/A	12,103 (803-150, 089)		N/A	391 (240-76,069)		N/A	15 (10-50)		N/A	1 (0-2)	
Value, range^e^		0-14	1-496	N/A	50-14,000,000		N/A	29-2,100,000		N/A	29-412,858		N/A	2-3538		N/A	0-10	

^a^ED: emergency department.

^b^ML: machine learning.

^c^The totals represent pooled data from all studies generated in that particular year.

^d^The number of studies represents how many studies the total was distributed across.

^e^Median (IQR) and range values were calculated based on individual study sample sizes; results reported by year are pooled.

^f^N/A: not applicable.

**Table 2:** Study characteristics by country.

Country	Studies (n=96), n (%)	Included EDs^a^		Initial sample			Included patients			Included visits			Included nurses			Studies using ML^b^ methods (n=39), n (%)
		Total (n=2438), n^c^ (%)	Studies (n=88), n^d^ (%)	Total (n=63,413,919), n^c^ (%)	Studies (n=79), n^d^ (%)	Total (n=1,804,813), n^c^ (%)		Studies (n=28), n^d^ (%)	Total (n=12,220,866), n^c^ (%)		Studies (76), n^d^ (%)	Total (n=3844), n^c^ (%)		Studies (n=9), n^d^ (%)		
United States	43 (44.79)	2008 (82.36)	39 (44.32)	36,528,693 (57.6)	35 (44.30)	916,955 (50.81)		12 (42.86)	4,986,560 (40.80)		34 (44.74)	3781 (98.36)		3 (33.33)	22 (56.41)	
Australia	31 (32.29)	404 (16.57)	27 (30.68)	23,110,878 (36.44)	24 (30.38)	1996 (0.11)		5 (17.86)	4,784,753 (39.15)		24 (31.58)	2 (0.05)		1 (11.11)	10 (25.64)	
Canada	5 (5.21)	7 (0.29)	5 (5.68)	6450 (0.01)	4 (5.06)	573 (0.03)		3 (10.71)	19,727 (0.16)		4 (5.26)	20 (0.52)		1 (11.11)	1 (2.56)	
Israel	3 (3.12)	3 (0.12)	3 (3.41)	1,586,760 (2.50)	3 (3.8)	648,294 (35.92)		3 (10.71)	1,361,455 (11.14)		2 (2.63)	0 (0)		0 (0)	2 (5.13)	
Great Britain	2 (2.08)	2 (0.08)	2 (2.27)	11,911 (0.02)	2 (2.53)	355 (0.02)		2 (7.14)	50 (0.0004)		1 (1.32)	4 (0.1)		1 (11.11)	0 (0)	
Brazil	1 (1.04)	1 (0.04)	1 (1.14)	499,853 (0.79)	1 (1.27)	0 (0)		0 (0)	499,853 (4.09)		1 (1.32)	0 (0)		0 (0)	1 (2.56)	
Switzerland	1 (1.04)	1 (0.04)	1 (1.14)	0 (0)	0 (0)	519 (0.03)		1 (3.57)	519 (0.004)		1 (1.32)	15 (0.39)		1 (11.11)	0 (0)	
China	1 (1.04)	1 (0.04)	1 (1.14)	44,237 (0.07)	1 (1.27)	295 (0.02)		1 (3.57)	295 (0.002)		1 (1.32)	0 (0)		0 (0)	0 (0)	
Spain	1 (1.04)	1 (0.04)	1 (1.14)	2080 (0.003)	1 (1.27)	0 (0)		0 (0)	1572 (0.01)		1 (1.32)	0 (0)		0 (0)	0 (0)	
Finland	1 (1.04)	2 (0.08)	1 (1.14)	52,032 (0.08)	1 (1.27)	0 (0)		0 (0)	42,247 (0.35)		1 (1.32)	12 (0.31)		1 (11.11)	0 (0)	
France	1 (1.04)	1 (0.04)	1 (1.14)	80,320 (0.13)	1 (1.27)	0 (0)		0 (0)	806 (0.01)		1 (1.32)	0 (0)		0 (0)	0 (0)	
Iran	1 (1.04)	1 (0.04)	1 (1.14)	537 (0.0008)	1 (1.27)	0 (0)		0 (0)	537 (0.004)		1 (1.32)	10 (0.26)		1 (11.11)	1 (2.56)	
South Korea	1 (1.04)	1 (0.04)	1 (1.14)	142,972 (0.23)	1 (1.27)	0 (0)		0 (0)	138,022 (1.13)		1 (1.32)	0 (0)		0 (0)	1 (2.56)	
New Zealand	1 (1.04)	1 (0.04)	1 (1.14)	1000 (0.001)	1 (1.27)	0 (0)		0 (0)	1000 (0.01)		1 (1.32)	0 (0)		0 (0)	0 (0)	
Portugal	1 (1.04)	1 (0.04)	1 (1.14)	599,276 (0.95)	1 (1.27)	235,826 (13.07)		1 (3.57)	0 (0)		0 (0)	0 (0)		0 (0)	1 (2.56)	
Portugal and United States	1 (1.04)	2 (0.08)	1 (1.14)	719,925 (1.14)	1 (1.27)	0 (0)		0 (0)	356,475 (2.92)		1 (1.32)	0 (0)		0 (0)	0 (0)	
Uganda	1 (1.04)	1 (0.04)	1 (1.14)	26,995 (0.04)	1 (1.27)	0 (0)		0 (0)	26,995 (0.22)		1 (1.32)	0 (0)		0 (0)	0 (0)	

^a^ED: emergency department.

^b^ML: machine learning.

^c^The totals represent pooled data from all studies generated in that particular country.

^d^The number of studies represents how many studies the total was distributed across.

### Study Objectives

The most common objectives for studies using triage narratives were to perform case identification (59/96, 61%), to use narratives as a predictor variable in ML models (21/96, 22%), and to use narratives for quality improvement (16/96, 17%; Table 3). Studies categorized with case identification as their primary objective sought to describe incidence or prevalence estimates or populations of interest. Studies that used narratives as a predictor variable predicted patient acuity scores, resource use, or specific diagnoses.

Quality improvement studies used triage narratives to increase clinician or system safety and were subdivided as pertaining to reliability, accuracy, and validity or safety and efficiency. Reliability and validity studies examined interrater reliability and were used to assess whether the triage classification matched specific populations with specific categorical assignments or triage acuity scores. Safety and efficiency studies examined narratives to improve data quality or reduce errors and effort (Table 3).

ML consisted of several models, and we used an inclusive approach by combining all ML, natural language processing, and other artificial intelligence models. We noted the frequency of ML use to be increasing and that ML was more frequently used in predictive studies (21/25, 84%) than in studies using narratives for case identification (17/58, 29%) or quality (1/13, 8%; Figure 2).

**Table 3:** Summary of study objectives.

Study category and types of papers in the category			Explanation
**Quality improvement**			
	Accuracy, validity, and reliability	Studies used triage narratives from previous ED^a^ visits as a research instrument. These studies would have nurses or physicians rescore visits and compare the scores to calculate the reliability, validity, accuracy, or interrater agreement of providers for specific triage systems [48,49,51,54,60-62].	
	Safety and efficiency	These studies examined quality as the completeness of triage data [47], as how time-sensitive presentations were handled at triage [63,64], and to identify or improve errors in acuity or category assignment [51,54,62,65,66]. Other studies focused on improving triage and measured the amount of duplicate or redundant information within triage narratives [67] or the efficiency [42,54,55], accuracy [55], and completeness [55,58,68,69] of narratives.	
**Case identification**			
	Syndromic classification	These studies had a primary objective of developing, describing, or comparing syndromic surveillance systems. These systems attempt to group all patients from a single large cohort into one of several broadly defined groups to assign a reason for visit category [38,42-44,50,52,53,70-76].	
	Estimate incidence or describe a population	Triage narratives have been used as an alternate means of identifying general or specific presentations. General grouping included cases related to drugs or alcohol [39,40,77-86], sports [69,87-90], motor vehicle collisions [41,90-93], mental health–related presentations, [94-100], environmental injuries [45,101-103], infections [104,105], assaults [106,107], and animal bites [45,108,109]. Narratives seem to be particularly good at identifying rare cases [107,110-113]. Narratives have also been used to provide granular data about patients, such as temporal information [114], to complete missed vitals [115] and to provide contextual details such as events leading to an injury [39,78,87,89-91,116].	
**Prediction**			
	Acuity or resource use	Predictions using triage narratives attempted to forecast the resource uses by patients in general [117] or addressed specific aspects of care, including the need for admission [118-122], triage acuity [57,59,123-126], length of stay [119], critical illness [124], and mortality, [57,127,128].	
	Specific diagnoses	Triage narratives were used as a covariate for machine learning models that predicted specific resource or admission needs. Admission destinations and resources of interest included advanced diagnostic imaging use [56,129,130], mental health admission [131], ICU^b^ admission [132], or neuro-intensive care unit admission [133].	

^a^ED: emergency department.

^b^ICU: intensive care unit.

### Descriptions of Triage Narratives

The quality and structure of the triage narratives used in each study were not clearly stated. Of the 96 studies included, only 30 (31%) described the narrative. The most common approach to describing narratives was a description of the triage narrative or of the keywords used to search within the narrative (Table 4).

**Table 4:** Descriptions of the structure of triage narratives.

Study, year	Description of the triage narrative^a^	Keywords, n^b^	Keyword topics
Travers and Haas [75], 2003	There was a description of the characteristic components of the narrative chief complaints that were not matched by machine learning: punctuation, truncations, modifiers, and qualifiers were discussed	N/A^c^	N/A
Chapman et al [104], 2004	N/A	5	The following fever-related keywords were used: “fever(s),” “Febrile,” “chill*,” and “low grade temp*”
Day et al [43], 2004	The mean length of the triage narratives was 14.6 (SD 7.9) words in each database	6	Shortness of breath and difficulty in breathing were examined
Thompson et al [38], 2006	The maximum allowable space for triage narratives was 40 characters	>100	Keywords for chest pain, syncope, earache, and others
Indig et al [39], 2010	The average triage note was 35 words (including abbreviations) per presentation; there was a secondary text field that was not discussed	>160	Drug and alcohol keywords
Bregman and Slavinski [109], 2012	N/A	2	Mammal bite–related terms and their associated animals were examined using the search terms “bite” and “animal”
McKenzie et al [108],2010	N/A	50	Work, worker, and work-related keywords and truncations
Vallmuur et al [79], 2013	N/A	18	Alcohol-related keywords
Mitchell and Bambach [91], 2015	N/A	32	Alcohol- and vehicular collision–related keywords
Luther et al, [101], 2016	N/A	1	Presentations with the keyword “heat”
Rahme et al [95], 2016	N/A	16	Suicide-related keywords were identified in both English and French
Whitlam et al [81], 2016	N/A	12	Alcohol-related keywords
DeYoung et al [40], 2017	N/A	>150	Cannabis-related keywords
Kondis et al [107], 2017	N/A	2	“Crying” and “fussy” were the search keywords reported; however, variations in these terms were also included (although not specified by the authors)
Harduar Morano et al [102], 2017	N/A	11	Heat injury–related keywords
Zhang et al [118], 2017	N/A	25	A list of keywords predictive of patient admission
Chu et al [110], 2018	N/A	1	“Headache”
Gligorijevic et al [117], 2018	N/A	24	Mixed keywords for a variety of presentations
Goldman-Mellor et al [131], 2018	N/A	8	Mental health and substance use–related keywords
Hargrove and Waller [92], 2018	N/A	23	Vehicle collision–related keywords
Nagabhushan and Webley [111], 2018	N/A	2	Specific chest pain feature keywords “ripping” and “tearing”
Chen et al [89], 2019	N/A	2	“Tramp” and “bounce” were specified, but other terms may have been used
Eley et al [90], 2019	N/A	14	Bicycle-related keywords
Marx et al [83], 2019	N/A	8	Marijuana-related keywords
Trivedi et al [93], 2019	N/A	3	Electric scooter–related brand names “bird” and “lime” as well as “scooter” were the keywords
Sterling et al [126], 2020	The mean length of triage narrative was 143.17 (SD 77.8) characters (excluding spaces) or 64.3 (SD 35.2) words	N/A	N/A
Vernon et al [41], 2020	N/A	3	Electric scooter–related keywords and their variations were searched. “Scooter,” “e-scooter,” and “electric-scooter” were offered as specific terms
Ivanov et al [125], 2021	The average number of clinical features per text entry was 12.79. There was no discussion about character or word counts	N/A	N/A
Rahilly-Tierney et al [86], 2021	N/A	3	“Heroin” and “overdose” were specified as inclusion terms and “detoxification” as an exclusion term; although there may have been additional terms included, they were not specified
Rozova et al [99], 2021	The average triage note was 127 characters long (notes with <30 characters were excluded)	40	Suicide-related keywords

^a^Studies reporting only the process of cleaning and normalizing unstructured narratives were not included.

^b^Variations in spelling, abbreviations, bigram duplications, and negation terms were counted if specified.

^c^N/A: not applicable.

There were 7 studies that described triage narratives [38,39,43,75,99,125,126]. The descriptions included the counts of characters and words used in the typical triage narrative. The length of the triage narrative entries in these studies ranged from 40 [38] to 127 characters [99] and 14.6 [43] to 35 words (including abbreviations) [39] (Table 4). One study described the narratives in terms of “clinical features” [125]. “Clinical features” in this study were Unified Medical Language System clinical terms that the authors derived using a natural language processing algorithm (C-NLP), but it is unclear how much these differ from their input data or whether they can be compared with those in other studies.

There were 27 studies that reported on the specific keywords that were present within the narratives [38-41,43,79, 81,83,86,89-93,95,99,101,102,104,107-111,117,118,125,126,131]. The number of keywords ranged from 1 [101] to >160 [39], with a median number of 11 (IQR 3-24.5) keywords reported (Table 4). However, 11% (3/27) of studies did not report the exact number of keywords used [38-40]. The authors reported the use of express keywords with correct spellings [86,93,101,107,109-111] as well as intentional variations such as misspellings, abbreviations, or truncations [39,40,81,92,108]. One of the studies searched for terms using keywords in 2 languages (English and French) [95].

In total, 9 studies reported the number of nurses who generated the narratives [38,48-55]. The total number of nurses whose documentation was assessed in these studies was 3844. The median sample size of nurses was 15 (IQR 10-50), and the sample size ranged from 2 [50] to 3538 [53]. These 9 studies represent only 3% of the total sample size (n=367,946). One of the studies reported on both the structure of triage narratives and the number of nurses included in the sample [38].

The most in-depth descriptions were provided by Travers and Haas [75], who explored triage narratives in depth by describing the structure of the narratives and regional variations. This 3-center retrospective cohort study used verbatim triage chief complaint narratives drawn from EDs in the United States. In a corpus of 13,494 unique chief complaint narratives drawn from 39,038 visits, they used manual and automated techniques to identify chief complaint concepts using the Unified Medical Language System data definitions. Concepts that were not readily classified using ML models were described in both form and function, and the authors detailed the function of the punctuation, acronyms and abbreviations, truncations, modifiers, and qualifier words used in triage narratives [75].

Although quality appraisal can be incorporated into scoping reviews [30], we did not opt to include one because our primary aim was to describe the literature rather than assess each study’s findings [27,28]. Consequently, we are limited to reporting that 8% (8/96) of the included studies used an Enhancing the Quality and Transparency of Health Research Network quality reporting guideline (Table 5). In total, 4% (4/96) of studies used reporting guidelines specifically for predictive models [62,99,124,129], and 1% (1/96) of studies reported using a quality framework to guide data cleaning and the protection of patient information [124].

**Table 5:** Studies that used reporting guidelines and the types of guidelines used.

Study, year	Reporting guideline	Guideline body
Chu et al [110], 2018	The RECORD^a^ statement	EQUATOR^b^ Network
Jones et al [82], 2019	The STROBE^c^ statement: guidelines for reporting observational studies	EQUATOR Network
Trivedi et al [93], 2019	The STROBE statement: guidelines for reporting observational studies	EQUATOR Network
Zhang et al [129], 2019	GRRAS^d^	EQUATOR Network
Joseph et al [124], 2020	(1) HIPAA^e^ Safe Harbor method and (2) The TRIPOD^f^ statement	(1) US Department of Health and Human Services and (2) EQUATOR Network
Cheung and Leung [62], 2021	GRRAS	EQUATOR Network
Lam et al [85], 2021	The RECORD statement	EQUATOR Network
Rozova et al [99], 2021	The TRIPOD statement	EQUATOR Network

^a^RECORD: Reporting of Studies Conducted Using Observational Routinely Collected Health Data.

^b^EQUATOR: Enhancing the Quality and Transparency of Health Research.

^c^STROBE: Strengthening the Reporting of Observational Studies in Epidemiology.

^d^GRRAS: Guidelines for Developing and Reporting Machine Learning Predictive Models in Biomedical Research.

^e^HIPAA: Health Insurance Portability and Accountability Act.

^f^TRIPOD: Transparent Reporting of a Multivariable Prediction Model for Individual Prognosis or Diagnosis.

## Discussion

### Principal Findings

We performed a scoping review to examine studies reporting on the structure and use of triage nurse narratives. Our search was systematic, used a prepublished protocol, and screened a significant number of studies published over a 32-year period. Our study protocol was registered and published and used standardized screening templates and data extraction forms [29]. Our search intentionally sacrificed specificity for sensitivity, including a substantial number of studies in keeping with the scoping review design. The volume of studies retrieved demonstrates that identifying triage narrative in academic literature is difficult and that straightforward ways of identifying pertinent studies are needed. Studies would be more readily identifiable if their keywords, titles, and abstracts were clear and consistent.

In addition to the triage narrative, we found that the most frequently reported data were patient age, sex, chief complaint category, discharge status, and triage acuity, similar to a 2020 systematic review of ML for clinical decision support in the ED [5]. Similar to other review studies, we found an increase in the number of studies conducted over time [3]. We found a sharp increase in the sample size of studies after 2008. Our findings also support that the studies using ML lag behind studies of health record data. However, we noted that this trend continued only until 2017, when ML became the most common approach reported in the literature. Wang et al [3] tabulated the top sources of electronic health record narratives and determined that the most common sources were discharge summaries (n=26, 45% of studies), progress notes (n=15, 26%), admission notes (n=9, 16%), operative notes (n=5, 9%), and primary care notes (n=3, 5%). We identified 5 studies [71,72,108,121,134] that used ML. ML studies were challenging to identify through structured searches. Similar to our review, Wang et al [3] determined that most studies were conducted in the United States. They identified fewer (3/263, 1%) studies from Australia. In comparison, our study identified that 56% (10/18) of the studies originated from Australia during the same period [50,108]. Our results differ in part because we did not restrict our search in the same manner as Wang et al [3], who explicitly examined ML, and rather focused on unstructured narratives as a primary search concept.

The previously discussed reviews and several other studies included in this review established that triage narratives can improve case identification when used in isolation or when added to diagnosis codes [22]. The use of narratives for these purposes was reported as a straightforward process in several studies that showed that their inclusion or exclusion can substantially impact the number of cases identified [72,78,79]. Refinement of these techniques may improve the sensitivity of searches and have significant impacts on disease prevalence estimates for diagnoses (eg, rare illnesses) that may not be well captured with administrative discharge codes, a common method for tracking population illnesses [113,135]. The methods used in keyword-based case identification studies are well positioned for implementation, given their clearly defined and reproducible methods and long history of being used for these purposes. Studies of disease prevalence were among the first to use narratives collected on a large scale [42,75]. The potential improvements to the sensitivity and specificity of case identification may justify the systematic review of the studies included in this review. In addition, future research could focus on clearly defining the improvements that narrative data analysis can offer to case identification studies.

There is a pressing need to collect nursing data [7], and triage has been identified as one of the most important areas for quality improvement [136]. Several studies have reviewed quality improvement efforts at triage [8] and called to include narratives in these efforts [137], but significant work is still needed. A renewal of early efforts to establish a minimum ED nurse data set [26] and efforts to create common definitions for narrative elements are needed [25], as is additional research to describe the structures of triage narratives in general. This work is required to determine whether there is a common structure in the data. Our results showed that even though 31% (30/96) of studies offered a description of narratives, only 1% (1/96) provided significant depth. A fulsome description is needed to ensure that triage nursing contextual data are not lost through text normalization (a typical early step in data cleaning for models), given that nurses use unique punctuation and abbreviations while recording triage narratives [75]. Finally, given the wide regional variations in the breadth and depth of information collected at triage, research is needed to identify the specific details that triage narratives should contain.

The Strengthening the Reporting of Observational Studies in Epidemiology and Reporting of Studies Conducted Using Observational Routinely Collected Data guidelines were published in 2007 [138] and 2015 [139], respectively. However, only 8% (8/96) of the studies reported using a reporting guideline, even though 86% (83/96) of these studies were reported after 2007. Recently published reporting guidelines such as the Transparent Reporting of a Multivariable Prediction Model for Individual Prognosis or Diagnosis [140] may contribute to more consistent reporting guideline use, and 2021 saw the highest (3/13, 23%) proportion of studies using a reporting guideline. The use of reporting guidelines will help reduce the heterogeneity noted in reporting.

### Limitations

In total, 3 concepts (emergency, triage, and narrative) were searched using an inclusive search approach, resulting in a substantial number of studies. The level of agreement during screening was fair, but it was likely reduced owing to the large number of studies reviewed and the need for full-text reading to determine whether the narrative was nurse generated. Future refinements to the search strategy may enable a less wide-reaching search, and more clearly defined methods to identify nurse-generated narratives may decrease the number of studies for screening. In addition, clear methods for identifying when narratives are generated by nurses may prevent researchers from pooling nurses’ triage narratives with narratives generated by other clinicians such as physicians, which may result in more studies being positively identified as originating from triage nurses.

### Conclusions

This review identified 96 studies that used triage narratives to achieve quality improvement, perform case identification, or make predictions about clinical outcomes. We have described how narrative use is changing to incorporate larger samples and more ML methods for interrogating the data. We have provided a strong argument that there is a considerable lack of research on the structure of triage narratives. Future research should not only focus on the outcomes of their study but also describe in detail the data sources used. Future researchers should strive to follow reporting guidelines to improve the quality of data reporting and increase the ability to pool and compare study findings. Emergency nursing scholars can encourage the national collection of triage data to allow comparison between regions if the common structures of data are better articulated.

## Appendix

Multimedia Appendix 1 Summary of the included studies.

Multimedia Appendix 2 Data items collected.

## Abbreviations

ED: emergency department
ML: machine learning
PRISMA-ScR: Preferred Reporting Items for Systematic Reviews and Meta-Analyses Extension for Scoping Reviews
