# Supplementary material for: The Use and Structure of Emergency Nurses’ Triage Narrative Data: Scoping Review
Source: JMIR Nurs. 2023 Jan 13;6:e41331. doi: 10.2196/41331 (PMC9883744; doi:10.2196/41331)
Supplement: Multimedia Appendix 1 [file nursing_v6i1e41331_app1.docx]

Multimedia appendix 1. Summary of included studies

| First author, Study year | Country | Narrative use | Study objective | Summary of findings |
| --- | --- | --- | --- | --- |
| Kabir, A. 1998.[47] | AUS | Quality improvement | To compare the completeness of documentation occurring with paper charting versus computer assisted triage. | This retrospective observational study of consecutive children presenting for acute asthma to an ED compared the degree of information gathered during computer assisted triage it to non-computer triage and physician documentation. Using descriptive statistics, and kappa values they reported on: visit characteristics, triage details, nursing observations, medical details, asthma severity, nursing observations, determining these were better recorded in the paper chart than the computer chart. |
| Beveridge, R. 1999.[48] | CAN | Quality improvement | To examine the reliability of the triage acuity assigned by different observers. | This retrospective analysis of routinely collected data with prospective rescoring for validations examined 50 patient records (10 from each acuity level) and re-scored them using summarized visit details that included: the presenting complaint, mode of arrival, vitals, and the verbatim triage narrative. Between and within group comparisons (nurses, physicians) were made using kappa coefficients, and 1 and 2-way ANOVA testing. Between group and within group acuity scores had high degrees of agreement, ANOVA testing showed that physicians assigned higher triage scores overall. |
| Goodacre, SW. 1999.[49] | GBR | Quality improvement | To perform triage quality improvement by examining interrater agreement on triage. | This retrospective used manual chart reviews to rescore triage visits and compared triage nurse accuracy. Full chart data (including triage narratives) were used to reassign triage acuity levels. Acuity scores were compared using Kappa, Sensitivity, Specificity, NPV, PPV, Sensitivity/recall and F-scores. Kappa scoring showed fair to moderate inter-rater agreement. Predictive power of scores did not improve with after developing of triage guidelines although there were modest improvements in agreement scores. |
| Aronsky, D. 2001.[42] | USA | Case identification | To reduce the frequency of free text triage chief complaints narratives by developing a list of coded chief complaints. | This was a three-step quality improvement project that used retrospective triage narrative data to develop a chief complaint category list, prospectively examined whether the list reduced the frequency of free text chief complaint documentation, and described staff satisfaction with the project. Using descriptive statistics and control charts the authors described the final complaint list, frequency of free text narrative charting, and responses to Likert scale questionnaire responses. The intervention resulted a final chief complaints category list that included 54 coded variables, free text charting decreased from 23% to 1%, the number of staff using free text decreased from 45% to 9%, and ED staff found the intervention beneﬁcial. |
| Burt, CW. 2001.[87] | USA | Case identification | To estimate and compare the incidence and characteristics of sports and non-sports-related injuries to USA EDs. | This retrospective cohort study used the triage narratives from visits identified using diagnosis codes to describe the accuracy of injury diagnosis codes and assign activities. They found that males were more than twice as likely to have sports related injuries in the 5-24year old patients’ group (48.2 versus 19.2/1,000 persons). Basketball and cycling were the most commonly noted activities associated with injuries. |
| Howe, A. 2002.[106] | GBR | Case identification | To identify victims of assault, to compare the number of identified cases to the number recorded by police, and to identify the location and method of assault. | This was a retrospective observational study of patients who presented to the ED after assault. The authors interrogated physician and triage nurse narrative fields using "assault" keywords to identify cases. Cases identified using narrative field searches were compared to those identified using hospital and police maintained categorical codes. Descriptive statistics and Mann-Whitney U were used to describe and compare: the number of cases identified, location of assault, method of assault, and rates of identification between groups and methods. 2.6% of ED patients presented after an assault34% of cases were identified by both triage nurses and physicians. Triage nurses identify 57%, physicians 77% of assaulted patients. Differences in detection were not related to the patient’s age or sex. |
| Begier, EM. 2003.[70] | USA | Case identification | To compare the triage narrative derived syndromic chief complaint code to discharge diagnosis codes. | This retrospective cohort study reviewed the syndromic codes that were assigned by two expert reviewers (using the triage narratives) to the recorded discharge diagnosis. The overall agreement for the syndromes of: “death”, “gastrointestinal”, “neurological” “rash”, “respiratory” “sepsis”, “unspecified infection”, and “other.” To the discharge diagnosis varied significant kappa values raged from 0.085 to 0.684 for different syndromes. |
| Travers, DA. 2003.[75] | USA | Case identification | To use machine learning models to build a concept-oriented set of ED nursing terminologies from triage narratives. | This was a prospective comparison of different natural language processing models that used retrospectively collected triage narratives to create triage "concepts". Expert evaluation was used to examine the model’s accuracy in mapping concepts to Unified Medical Language System concepts. the authors detailed the steps used to normalize data and used descriptive statistics to describe the number of concept matches made after each round of narrative data normalization. |
| Chapman, WW. 2004.[104] | USA | Case identification | To compare the sensitivity and specificity of three machine learning models for detecting fever from triage narratives. | This study used retrospective electronic health record data to compared the sensitivity and specificity of three machine learning algorithms for detecting fever from triage and discharge narratives. Using patients identified with an ICD-9 discharge diagnosis of fever models were trained to use narratives and coded chief complaints to detect fever. Sensitivity, specificity, and positive likelihood ratios were used to compare different models to expert clinician ability to detect fever. Machine learning models using narratives were most sensitive, models using keywords the most specific when compared to expert clinicians. |
| Day, FC. 2004.[43] | USA | Case identification | To use word clusters to automatically link free text with reason for visit categories based on ICD codes. | This was a retrospective of ED data from a state database that used word clusters to automatically link triage narratives with reason for visit categories. Using patient age, sex, free text chief complaint, and diagnosis (based on ICD codes) cases were identified. Descriptive statistics were used to describe salient keywords identified, the character count of narratives, and the percentages of correctly coded chief complaints by age. Sensitivity/specificity analyses were used to build inclusion and exclusion keyword clusters and to compare clusters to ICD code categorization. |
| Mikosz CA. 2004.[134] | USA | Case identification | To compare the agreement between two coding programs and their associated free text triage notes. | This was a retrospective cohort study that compared the triage chief complaints assigned to free text narratives by two different machine learning algorithms. Overall agreement varied by syndrome (Kappa = .419 - 0.711). |
| Baumann, MR. 2005.[60] | USA | Quality improvement | To determine the reliability and validity of ESI for pediatric populations. | This study retrospectively examined triage EHR data and had to assess the reliability and validity of ESI scores for pediatric patients. Patient and visit characteristics were described. The relationship between resource use and ESI scores were examined, inter-rater agreement of re-triaged scores. Weighted agreement ranged from good to excellent. Hospitalization, ED length of stay, and resource utilization were strongly associated with ESI category. |
| Chapman, WW. 2005.[71] | USA | Case identification | To evaluate a machine learning algorithm’s ability to classifying free text data into syndromic categories for surveillance purposes. | This retrospective observational study examined EHR data to compare physician "gold standard" coding to a machine learning model’s ability to classifying free text data into syndromic categories. machine learning classifier AUC scores ranging from 0.95 to 1.0. |
| Chapman, WW. 2005.[72] | USA | Case identification | To examine a machine learning programs accuracy in classifying syndromic categories and to determine if the program can be used to examine chief complaints in triage narratives. | This retrospective observational study used ICD codes from EHR data to determine if a machine learning model could accurately classify syndromic categories using triage chief complaint narratives. The models accurately classified 92% of neurologic and respiratory syndromes and 99% of botulinic syndromes. The models sensitivity ranged from 30% to 75%, with specificities greater than 90%, and positive predictive values of 12% to 44%. |
| Muscatello, DJ. 2005.[73] | AUS | Case identification | To test a syndromic surveillance system based on routinely ED data. | This study describes the development and implementation of an automated near real-time syndromic surveillance using routinely collected ED data. The evaluation compares a machine learning model that examined patient demographic, presenting complaints, and triage narratives to physician-assigned ICD and departure codes. The machine learning model assigned syndromic classifiers to a greater proportion of ED visits 93% (range: 58% – 100%) compared to the availability of physician completed ICD codes 76% (range: 68% – 86%). During the assessment period the EDs had a median of 997 (range: 941–1077) visits per day and there were no outbreaks recorded. |
| Thompson, DA. 2006.[38] | USA | Case identification | To use word clusters to automatically link free text with reason for visit categories. | This retrospective study used EHR data to automatically link free text triage narratives word clusters with reason for visit categories based on ICD codes using a machine learning model. Demographics and visit characteristics are described; 87.5% of visits were successfully classified. |
| Gillam, C. 2007.[116] | AUS | Case identification | To examine an injury surveillance system and compare nurse injury coding to the narrative text in order to determine levels of agreement and sources of error. | This study assessed the validity of an injury surveillance system using EHR data for all patients presenting with injuries. Researchers extracted narrative description of the circumstances leading to the presentation, intent, and cause using triage nurse narratives. Agreement between the triage nurses’ coding and those assigned using the narrative were compared for each data element. Of the included cases (n=419) triage nurses and expert agreement was 91.9% for intent and 79.2% for cause. |
| Indig, D. 2008.[77] | AUS | Case identification | To compare provisional medical diagnosis and nursing triage text for detecting alcohol-related ED presentations and to explore visit related data to examine why alcohol-related presentations are under-detected. | This study compared two methods for detecting alcohol-related ED presentations: diagnosis codes and nursing triage text, and compared patient and visit characteristics examine missed formal diagnoses and describe why alcohol-related ED presentations are under-reported. Triage free-text fields, retrieved from a surveillance database, were searched using alcohol-related terms. Multivariate Log regression analysis of demographics, diagnostic info, service delivery characteristics, and free text (triage) was used to identify factors predictive of medical versus triage identified cases. Approximately 4.5% of ED presentations were alcohol-related, 24% were identified through diagnostic codes and by triage narrative. Presence of diagnostic coding was more for patient arriving by ambulance or those with signs of aggression; and less likely for patients with injuries. |
| Irvine, AK. 2008.[114] | USA | Case identification | To describe a program designed to extract temporal information from triage notes. | This study reports on a program designed to extract temporal information from triage notes. It prospectively compares a machine learning model to an expert reviewer’s ability to extract information from triage narratives. The most common class of time statements were relative (e.g., 1 week ago), there was perfect agreement between time coders, decision tree out-performed models outperformed naive bayes machine learning in precision and recall while assigning data. |
| Indig, D. 2009.[78] | AUS | Case identification | To examine different methods for detecting alcohol-related ED presentations and to compare these patients to ED patients identified as risky drinkers by a questionnaire. | This multiple step study compared different methods (triage narratives, ICD codes, and questionnaire) for identifying alcohol-related ED presentations and to compare the characteristics of each group. Proportions of patients identified using each method were compared and multivariate logistic regression was used to determine which characteristics are predictive of alcohol related presenting complaints. ICD codes had a higher specificity and lower sensitivity than triage narratives for identifying total number of presentations and percentage of risky drinkers presenting for an alcohol related complaint. |
| Mitchell, R. 2009.[88] | AUS | Case identification | To identify sports injuries from triage narrative text. | This retrospective cohort study sought to identify sports injuries using triage narratives from a national ED database. A 5% sample of cases flagged using keywords were manually audited to confirm true and false positive rates for each keyword used. Within the sample some sports had higher precision (Ex. Rugby) than others (Ex. Squash). |
| Indig, D. 2010.[39] | AUS | Case identification | To use triage narrative to detect drug and alcohol related presentations and describe their service delivery characteristics. | The purpose of this study was to use retrospective EHR data (triage narratives) to detect drug and alcohol related presentations and describe their service delivery characteristics. The triage narrative identified 90% of drug and alcohol related presentations, physician assigned ICD detected 21% of drug and 25% of alcohol related visits. |
| Wagholikar, AS. 2011.[50] | AUS | Case identification | To use a rule-based approach to classify unstructured triage narratives into symptom groups. | This study compared the sensitivity and specificity of a text classifying algorithm to expert categorization in identifying chest pain from triage narratives. the algorithm was 99.3% sensitivity, 80.0% specificity, and had an F-score of 0.9 when compared to expert assignment for identifying chest pain. |
| Bregman, B. 2012.[109] | USA | Case identification | To use triage narratives to identify and characterize visits to EDs for animal bites. | This retrospective cohort study identified animal bite rates using triage narrative searches for the word "bite". Over 6,000 animal bite visits were identified on a yearly basis. 70% of animal bites were from dogs, 13% from cats; the characteristics of visits were similar to other surveillance systems. |
| Grossmann, FF. 2012.[51] | CHE | Quality improvement | To examine the validity, reliability, and accuracy of triage acuity scores for adult patients and to describe the reasons for acuity errors. | This prospective cohort study examined triage data (original narratives) retrieved from hospital records of patients greater than 65 years of age. Visits were rescored by two blinded triage experts for agreement, acuity scores were compared to resource use, length of stay, disposition, need for lifesaving intervention, and mortality. Agreement between original score and expert re-triage was high (weighted kappa=0.934, 95% CI 0.913 to 0.954). Under triage occurred in 117 cases, with inaccurate interpretation of vital signs being the most common cause. |
| Malmström, T. 2012.[52] | FIN | Case identification | To classify use free text and diagnostic codes to produce ED specific complaint classification codes. | This retrospective descriptive study with prospective validation and focus group interviews. The first phase described the process used to produce an algorithm that groups complaint categories using triage narratives and diagnostic codes. The second phase prospectively examine the implementation of the algorithm. The final phase was evaluated by an expert panel. The presenting complaints summarized from free text fields were similar to those of the algorithm. The algorithm included 89 presenting complaints and ED staff found it easy to use. |
| McKenzie, K. 2010.[108] | AUS | Case identification | To compare different methods for identifying potential work-related injury cases using textual data and to compare the predicative power of each method to coded injury surveillance data for work related injuries. | This retrospective cohort study described and compared methods for identifying potential work-related injury cases using triage narratives. It examined the sensitivity, specificity, and positive predictive value of different keywords used in text interrogation against coded injury surveillance in a national database. |
| Rhea, S. 2012.[103] | USA | Case identification | To use routinely collected ED data to characterise heat related injury visits. | This retrospective cohort study used routinely collected ED data and weather data to characterize heat related injury visits. Visits containing a heat-related illness ICD-9 code has their chief complaint and triage narrative data examined for potential exposure categories. Descriptive statistics were used to describe visit characteristics and distributions, patient characteristics, and regional temperatures. Visits were most likely in June, Heat related visits increased by 1.4 for each 1-degree Fahrenheit) increase from 90F to 98F and by 15.8 for each 1F increase from 98F to 100F. Distributions of causes (sport or work) were age related. Admission rates increased after age 65. |
| Genes, N. 2013.[115] | USA | Case identification | To transform free text vital sign data into numeric scores. | This retrospective cohort and prospective derivation study examined an algorithm’s ability to transform narrative triage vital sign data into numeric scores. 98% of values assigned from narrative data had perfect quality scores. Errors rates did not increase with periods of high triage throughput. |
| Mosley, I. 2013.[63] | AUS | Quality improvement | To describe the triage patterns associated with using rapid care protocols in patients who present within two hours of acute stroke symptom onset. | This retrospective observational study investigated factors associated with triage acuity assignment and initiation of stroke protocols for patients presenting to the ED within 2 h of symptom onset. Using EHR data researchers identified patients with an ED diagnosis of stroke or TIA and using logistic regression examined demographic and situational factors (including triage narratives) associated with a triage category of 1 or 2. 94% of patients diagnosed as a stroke or TIA who presented within 2 hrs were correctly identified. In all cases not correctly identified the patient was diagnosed with intracranial haemorrhage. |
| Vallmuur, K. 2013.[79] | AUS | Case identification | To compare different approaches for identifying alcohol involvement in youth who present to the ED for injuries and to describe the text used to triage these patients. | This retrospective cohort study compared methods for identifying alcohol involvement in injury-related youth ED presentations. Using triage narrative keywords and ICD codes researchers searched triage narratives to determine which keywords were most frequently used and identify visit characteristics and patient demographics associated with alcohol use. 6.4% of injury presentations had documented of alcohol involvement, patients 18 to 24-year-old, females, indigenous youth, Saturday or Sunday presentations, and presentations between midnight and 5 am were more likely to be alcohol related. Generic alcohol terms such as ethanol or alcohol were the most common terms used in the triage narrative. |
| Haas, SW. 2014.[53] | USA | Case identification | To develop and test a syndromic classifier using triage notes and chief complaints. | This was a retrospective cohort and model validation study. It used previously collected and classified syndromic database data to update existing syndrome definitions. Using triage notes and coded surveillance data that was manually classified by three clinicians’ researchers used the chief complaint, triage narrative, diagnosis, vital signs and admission status to determine if the updated definitions correctly classified visits into syndrome groups. Descriptive were used to describe triage narratives. Agreement was measured using kappa statistics. Performance measured using sensitivity and specificity. The triage narratives contained an average of 22 words in the initial dataset; 25 words (151 characters) in the follow-up dataset. Kappa for the two studies were 0.76 and 0.82 respectively. The new definitions improved the sensitivity and specificity for each syndromic cluster. |
| Liljeqvist, HTG. 2014.[94] | AUS | Case identification | To compare the accuracy of different methods of identifying mental health-related ED visits. | This retrospective cross-sectional study compared the accuracy of using different classification methods identify mental health-related visits using EHR data. The accuracy of different methods of identifying models: triage narrative data, ICD codes, and SNOWMED Codes were evaluated by comparing their case identification rates to expert coding using descriptive statistics, kappa scores, and by calculating each model’s sensitivity and specificity. Agreement between clinician’s classification and model classification ranged from moderate to almost perfect (Kappa 0.73, 95% CI 0.58 - 0.87), models were moderately sensitive (68% 95% 95% CI 46%-84%) and highly specific at (99%, 95% CI 0.98-0.997). Positive predictive value was 81% (95% CI 0.57 – 0.94) and negative predictive value was 98% (95% CI 0.97-0.99). |
| Rhea, SK. 2014.[45] | USA | Case identification | To describe the incidence of animal bite injuries. | The aims of this retrospective cohort study were to describe the incidence of animal bite related ED visits. Using surveillance codes, chief complaint codes, ICD codes, and triage notes researchers coded visits by animal (bat, bear, beaver, chipmunk, coyote, fox, groundhog, hedgehog, mole, opossum, otter, prairie dog, raccoon, skunk, squirrel, wolf, or woodchuck) and determined the incidences rates for each. By age of 10, patients had a 1 in 50 risk of dog bite injury. Dog bite rates were highest for patients ≤ 14 years of age; cat bites and scratches were most common in patients > 79 years of age. The lifetime risk of a cat bite or scratch (requiring ED care) was 1 in 60. Postexposure rabies prophylaxis was given to 1,664 of 38,971 visits. |
| Handly, N. 2015.[121] | USA | Predict outcomes | To determine whether machine learning algorithms using coded triage chief complaint data outperformed algorithms that did not in predicting hospital admission. | The objective of this two phase (derivation and validation) retrospective cohort study was to determine whether machine learning models using coded triage chief complaint data outperformed algorithms that did not in predicting hospital admission. In the derivation phase a machine learning model identified 213 chief complaint codes from the triage narratives, and identified other predictor variables such as: age, sex, race, visit characteristics (arrival time and day) and triage acuity. In the validation phase the sensitivity/specificity of models without and with coded triage complaints were 64.0% (95% CI 63.7–64.3)/87.7% (95% CI 87.4–88.0), 59.8% (95% CI 59.5–60.3%)/91.7% (95% CI 91.4–92.0) respectively. In the derived phase the sensitivity/specificity models without and with coded triage data were 60.7% (95% CI 60.4–61.0)/87.7% (95% CI 87.4–88.0) and 59.8% (95% CI 59.5–60.3)/90.6% (95% 95% CI 90.3–90.9) respectively. |
| Hides, L. 2015.[80] | AUS | Case identification | To describe the characteristics of alcohol related ED presentations. | This retrospective cohort study describes the visit characteristics of alcohol related injury presentations in the ED. Keyword searching of the triage narratives identified 7,381 of 12,264 cases. Descriptive statistics were used to report frequencies and proportions. Chi-square values were used to compare groups according to use of alcohol, demographics, location of injury, visit characteristics (time/date), and triage urgency. The patient cohort identified using triage narratives accounted for 38% of alcohol related injury presentations. The most common cause of injuries was falls and violence in males; intoxication and self harm in females. |
| Mitchell, RJ. 2015.[91] | AUS | Case identification | To use surveillance database data to identify injury risk factors of road users by age group. | This retrospective interrogated a nationally injury surveillance data base with various keywords then manually reviewed one percent of identified cases to estimate the likelihood of true positives. They also compared collision characteristics (e.g., vehicle speed) and injury risk factors (e.g., non-restraint use) by age group and road users’ type (i.e., motor vehicle drivers, motor vehicle passengers, motorcyclists, pedal cyclists and pedestrians). Descriptive statistics were used to describe demographics, and number of cases identified with each keyword. |
| Gray, SE. 2016.[69] | AUS | Case identification | To examine the completeness of triage data collected for fitness-related injuries. | This retrospective cohort study of the Victorian Emergency Minimum Dataset used fitness facilities injuries to examine the completeness of coded data. Using an anonymized ten-year sample of data on patients the authors created new variables (degree of specificity, location, activity, and product) using narrative data. Descriptive statistics were used to compare the frequency of each new variable. Cross-tabulations were used to compare the narrative coded data to existing categorical codes. Of the 2,936 identified cases: two percent could not have any additional information coded, and 95.8 % had at least one piece of information missing. There was injury type and body region coding in 92.6 and 96.6 % of cases, but only in 27.1 and 75.4 % of narratives. The causal variable was specifically coded in 47. 7 % of cases and in 45.9 % of narratives. |
| Luther, M. 2016.[101] | AUS | Case identification | To compare triage visits to environmental data to determine whether a change in heat warning threshold would change the predictive power of a surveillance tool. | This retrospective chart review of electronic hospital records used keyword searches of triage narratives to identify heat-related presentations. Heat-related presentation volumes were compared to the mean temperature of the three preceding days. Patient demographic and visit related details where collected. Using these data, the authors determined that temperatures above 30C were associated with increased risks, that extremes of age and outdoor activity was associated with increased risk. The symptom most strongly associated with presentations was syncope. |
| Rahme, E. 2016.[95] | CAN | Case identification | To describe the visit characteristics of patients who present to the ED for a suicide attempt, to identify factors associated with admission, and to validate ICD-10 codes as a method to detect suicide attempts in routinely collected data. | This retrospective cross-sectional study used ICD codes and triage narratives to identify patients who were seen in the ED and hospitalized following a suicide attempt. The purpose of the study was to describe patient and visit characteristics, identify factors predictive of need for hospitalization, and to compare the number of cases identified using ICD (10) codes for ‘‘intentional self-harm". 5746 cases were identified; 369 were fully reviewed, 281 (76%) were identified using the triage keyword method. Of these, 176 were treated in the ED and 193 required hospitalizations, 46% of patients received an ICD intentional self-harm code. 46% of cases were poisonings (the most frequent method), half of patients were less than 34 years old, 53% were female, and 75% had a previous history of mental health complaints. |
| Whitlam, G. 2016.[81] | AUS | Case identification | To evaluate the precision of routinely collected ED data in identifying acute alcohol-related harms. | This retrospective study randomly sampled 1,000 ED visits with an ED diagnostic code of alcohol harms and reviewed the triage narrative to confirm the diagnostic code and classify the visit as ‘acute’ or ‘chronic’ harm. Predictive factors for alcohol related presentations (ex, age, sex, time of arrival, etc.) and the predictive value of triage narratives were for acute harm were calculated. The PPV of diagnostic codes for acute alcohol harm was 53.5%. Variables predictive of acute harm were: ambulance arrival (aOR = 3.4, 95% CI 2.4–4.70, age (12–24 vs. 25–39 years: aOR = 3.4, CI 2.2–5.3), admission (not admitted) status (aOR 2.2, 95% CI 1.5–3.2), and arrival time (between 2200hrs and 0600hrs) (aOR 2.1, 95% CI 1.5–2.8). |
| Berendsen Russell, S. 2017.[67] | AUS | Quality improvement | To describe a method for reducing duplicate and redundant clinical terms in ED documentation systems. | This retrospective study describes a method for reducing duplicate and redundant information in ED electronic charting systems. Using narratively entered chief complaint data researchers identified 64,849 unique complaints from 1.7 million visits. Of these complaints 450 terms were used more than 100 times. Of the terms used 177 (39.3%) matched current definitions. The authors categorically grouped complaints into alternate clinically meaningful groups: cardiovascular (chest pain, arrhythmias), respiratory (shortness of breath, cough), gastrointestinal (abdominal pain, vomiting), and injury (fractures, trauma). |
| DeYoung, K. 2017.[40] | USA | Case identification | To use structured and free-text emergency data to develop and validate a syndromic definition for marijuana related ED visits. | This retrospective cohort study used structured triage variable and unstructured narratives to develop a syndromic case definition for marijuana related ED visits. Different ways of identifying cases were compared (triage narrative, discharge codes, discharge narrative, chief complaint, clinical impression) were compared to expert review (gold standard). There were 6 major keywords identified that yielded PPV of 82.5-100% PPV for case identification in different clinical fields. The triage notes and chief complaints have PPVs of 88.0 and 82.5%, respectively. |
| Horng, S. 2017.[105] | USA | Predict outcomes | To describe the utility of free text data in identifying patients with suspected infections. | This retrospective study describes a method for reducing duplicate and redundant information in ED electronic charting systems. Using narratively entered chief complaint data researchers identified 64,849 unique complaints from 1.7 million visits. Of these complaints 450 terms were used more than 100 times. Of the terms used 177 (39.3%) matched current definitions. The authors categorically grouped complaints into alternate clinically meaningful groups: cardiovascular (chest pain, arrhythmias), respiratory (shortness of breath, cough), gastrointestinal (abdominal pain, vomiting), and injury (fractures, trauma). |
| Kuramoto-Crawford, SJ. 2017.[96] | USA | Case identification | To evaluate and describe whether unstructured triage chief complaint data can identify suicide-related ED visits. | This retrospective study examined all ED visits with an unstructured narrative chief complaint or diagnosis that mentioned a suicide related term. These cases were examined and numbers of cases identified using each field were compared to describe the patient characteristics and numbers of cases identified using each method. The numbers of cases identified by method did not vary according to patient sex or age. Chief complaint narratives identified 62% of cases, discharge diagnoses identified 38% of cases. |
| Kondis, J. 2017.[107] | USA | Case identification | To determine the incidence of abuse related fractures for infants with prior ED visits for fussiness. | The aim of this retrospective cohort study was to determine the incidence of diagnosing fractures suggestive of abuse in infants who presented to the ED with "fussiness". Triage key word searches of electronic health records and imaging reports from radiology databases infants younger than 6 months were used to identify cases. Descriptive statistics were used to describe the frequency and distribution of ICD codes, chief complaints, presence of fracture, intervals between visits, outcomes of radiology variables in 18 patients. Of the 16 patients with fractures suggestive of abuse: the mean age was 2.5 months (SD=1.2). The mean interval between the initial and second presentation was 27 days. The most common diagnoses were multiple fractures, extremity fractures, and rib fractures. |
| Harduar Morano, L. 2017.[102] | USA | Case identification | To improvements the current heat-related illness syndrome classification definition. | This retrospective validation study's aim was to update the heat-related illness syndrome definition used in a syndromic surveillance database. Using heat-related illness diagnostic codes and triage narrative keywords researchers identified and characterized: the presentation characteristics of heat-related illnesses, compared the numbers of cases identified using existing and updated definitions, compared the PPV for each search strategy, and described the correlation coefficient for different data sources (triage note, chief complaint, and ICD codes). The existing definition identified 8,928 ED visits; the updated definition identified an additional 598 ED visits. Of the triage narrative (keyword) identified visits (n=4,006), 3216 (80.3%) were identified using the following terms: “heat ex” (n=1674, 41.8%), “overheat” (n=646, 16.1%), “too hot” (n=594, 14.8%), and “heatstroke” (n=302, 7.5%). |
| Zhang, X. 2017.[118] | USA | Predict outcomes | To compare the predictive power of machine learning models that use natural language processing (using triage narratives), to those that do not, in predicting admission to hospital. | This cross-sectional study examined a probability sample of nationally representative ED data ED from a two-year period. Data available at triage (including the triage nurse narrative) was used to develop machine learning models (with and without unstructured narrative data) to predict hospital admission. Variables derived using natural language processing from the unstructured triage narrative were used to explain 75% of the variance in hospital admission. machine learning models using structured data only had AUCs scores of 0.823-0.824 (95% CI 0.817-0.829 to 0.818-0.830) For logistic regression and neural-network models, respectively. machine learning models using unstructured data alone had AUC of 0.742 (95% CI 0.742-0.764). Models using both types of data had AUCs of 0.844 to 0.846 (95% CI 0.836-0.852 to 0.839-0.853) for logistic regression and neural network models, respectively. |
| Chu, KH. 2018.[110] | AUS | Case identification | To compare the incidence and outcome of intracranial hemorrhage in the general population and in ED patients presenting with headache. | This retrospective cohort study's aim was to describe the proportion of patients who presented to the ED with headache who received a diagnosis of subarachnoid hemorrhage and to compare it to the incidence in the general population. Using a regional ED database and vital statistics bureau birth, death and marriage registry. Patients with an ICD codes for subarachnoid hemorrhage, triage narrative including headache keywords, and a death registry subarachnoid hemorrhage cause of death were compared. Descriptive statistics were used to describe ICD codes, mortality, demographics, and incidence. The incidence of subarachnoid hemorrhage was 9.9 (95% CI 9.5–10.4) per 100,000 person-years. The in-hospital mortality rate was 23.8% (95% CI 22.0–25.8%). 1.9%, (95% CI 1.8–2.0) of ED patients with headache were diagnosed with subarachnoid hemorrhage. |
| Gligorijevic, D. 2018.[117] | USA | Predict outcomes | To develop a machine learning model that can predict resource requirements for ED patients | This retrospective cohort study used the triage narratives from ED visits to develop a machine learning model to predict resource utilization for ED patients. Visit characteristics, patient characteristics, vital signs, and insurance status were used as inputs for the machine learning model. Using these combined sources, the machine learning model achieved an AUC of 88% for identifying resource intensive patients, an accuracy of 44% in predicting the category of resources, and was 16% more accurate than nurses in its predications. |
| Goldman- Mellor, S. 2018.[131] | USA | Predict outcomes | To describe presenting complaints syndrome classifier and to examine its ability to predict mental health diagnoses using discharge ICD codes. | This retrospective cohort study describes a presenting complaints syndrome classifier and its ability to predict mental health diagnoses using diagnosis codes. A machine learning model was used to automatically classifies free text into chief complaints to identify cases as belonging to a syndrome group. Syndrome groups were compared to diagnosis codes. Agreement between the machine learning model and discharge codes and the ability to identify cases was high (Kappa=0.92; Sensitivity=100%, Specificity=98.6%). |
| Hargrove, J. 2018.[92] | USA | Case identification | To describe the effects of different case definitions on identifying motor vehicle crash injury cases. | This retrospective cohort study used triage narratives to automatically classify patients presenting complaints into mental health syndromes. The authors compared discharge ICD codes to the classifier models identified cases in order to: identify cases, describe the typically presenting complaints in these patients, and to examine the classifier model’s ability to predict mental health diagnoses. Descriptive statistics were used to characterize presentations. The classifier model has excellent agreement with the ICD identification approach (Kappa=0.92), 100% sensitivity, and 98.6% specificity in identifying cases. |
| Hendin, A. 2018.[65] | CAN | Quality improvement | To describe the outcomes of patients older than 65 years who received lower acuity scores. | This retrospective validation study's objective was to describe and compare different motor vehicle collision case definitions. Using existing motor vehicle collision codes, triage texts suggestive of motor vehicle collision, or a combination of both researchers described demographic variables (sex and age), arrival means (ambulance, walk-in, other), insurance status (private insurance, Medicare, Medicaid, self-pay, or other), disposition (home, admitted, transferred, left without being seen, died). Patients identified using motor vehicle collision codes were more likely to be male, arrive by ambulance, and be admitted when compared to the other search methods. |
| Nagabhushan, M. 2018.[111] | USA | Case identification | To determine how frequently patients with confirmed acute thoracic-aortic dissection present to the ED with chest-pain described as “ripping’ or “tearing”. | This retrospective cross-sectional study examined emergency records to estimate the prevalence of chest pain described as “tearing” or “ripping” by patients who received a diagnosis of aortic aneurism rupture. Complete review of the emergency records (including triage narratives) failed to identify any cases in which the patients used “ripping” or tearing” descriptors. The authors suggest that the complaints which have been historically held as prototypical may not be commonly used by patients. |
| Petruniak, L. 2018.[64] | CAN | Quality improvement | To describe the variables associated with triage acuity classification in patients with sepsis. | This retrospective cohort study described patient and contextual variables associated with high- versus low-acuity triage scoring in patients diagnosed with sepsis. Patients admitted with sepsis, severe sepsis, or septic shock (ICD codes) had their triage records examined. Triage narratives were examined and variables such as: unwell appearance, communication barriers, number of comorbidities, number of prescription medication, and place of residence were assigned and used as variables in addition to: CTAS score and ED census. Logistic regression found communication barriers and cognitive impairment (OR 5.7; 95% CI 2.15-15.01), acute confusion (OR 3.4; 95% CI 1.3-8.2), unwell appearance (OR 3.4; 95% CI 1.7-7.0), and hypotension (OR=0.98; 95% CI 0.96-1.0) predictive of higher acuity classification. ED census, heart rate, respiratory rate, and temperature were not predictive of triage acuity assignment. |
| Rice, BT. 2018.[76] | UGA | Case identification | To develop and validate a chief complaint list for ED visits in Uganda. | This retrospective cohort study developed and validated a chief complaint list for emergency care in Uganda. Using triage narratives from consecutive visits to a rural ED authors developed groups and compared rater assignment of the derived categories. 555 chief complaints were used to initially categorize 95.8% of visits. This complaint list was further refined using a consensus process to yield a longlist of 451 total complaints and shortlist of 83 complaints. Interrater reliability using the shortlist was of complaints was 71.5% agreement, (kappa of 0.70), over 80% of all visits were categorized into 24 primary complaints. |
| Dehgani Soufi, M. 2018.[54] | IRN | Quality improvement | To design and evaluate a clinical decision support system that can be used at triage to improve the speed and accuracy of triage acuity assignment. | This multi-step study used observations, unstructured interviews, and survey data to design a clinical decision support tool and a crossover intervention to validate the tool. Keywords from triage narratives were used as predictor variables in a machine learning model that achieved an accuracy rate of 99.44% in test data, and helped improve the completeness of documentation from 76.72% to 98.5%. |
| Chen, M. 2019.[89] | AUS | Case identification | To compare the injury patterns and treatment costs in patients injured on trampolines at commercial and residential locations. | This retrospective cohort study compared injury and treatment patterns for trampoline related injuries that occurred at home versus a commercial trampoline park. The authors searched the triage narratives from a children’s hospital over a one-year period using trampoline specific keywords to identify cases. Demographics, incident location, injury pattern, and cost data were derived from visit case notes, imaging reports, and surgical reports comparisons were made between injuries sustained at home versus a commercial facility. of the 392 cases identified 68.9% occurred at home; 19.4% were from a commercial location. The median age of those injured at home was significantly younger than commercial locations (5.6 vs. 12.8 years; P < 0.001), There were significantly more females injured at commercial facilities than at home (61.8% vs. 48.2; P = 0.03). 27.3% of injuries resulted from a fall from the trampoline. Fractures were the most common injury (39.5%); 17.4% required admission, and 12.8% surgery. |
| Choi, SW. 2019.[59] | KOR | Predict outcomes | To develop and evaluate machine learning models to predict triage acuity. | This retrospective cross-sectional study trained and compared machine learning models to triage acuity levels using categorical data, free text triage narratives only, and both categorical data and triage narratives. Categorical data included demographics (sex and age), arrival characteristics (date/time, coded chief complaint, means of arrival, vital signs. The free-text triage narrative was an unstructured nursing note, one to three sentences in length that summarized the reason for presenting to the ED. The AUC of the model using categorical data was greater than models trained on free-text data only, the machine learning models with the highest AUC were trained on the entire dataset. |
| Eley, R. 2019.[90] | AUS | Case identification | To describe characteristics of patients presenting to the ED for bicycle collisions. | This retrospective cohort study used triage narratives to characterise bicycle collision characteristics. Researchers used keywords to identify cases and derive injury patterns, object collided with, and trends over time. The authors describe visit demographics and typical injury patterns. |
| Greenbaum, NR. 2019.[55] | USA | Quality improvement | To describe the effects of a machine learning driven autocomplete on the efficiency and quality of triage documentation. | This multi-phase mixed-methods study used: a retrospective cohort to derive a triage narration ontology, a prospective quality improvement method to implement of a machine learning driven autocomplete function into triage documentation, and a before-after assessment of documentation quality (defined as the frequency in which nurses needed to use unstructured data fields). The authors described patients and clinical characteristic. The machine learning autocompletes improved documentation completeness and data quality (based on expert consensus), while simultaneously reducing the numbers of keystrokes needed to document a triage assessment. |
| Jones, R. 2019.[82] | AUS | Case identification | To analyze methamphetamine related ED presentations. | This retrospective observational study analyzed methamphetamine related ED presentations in patients aged 13-59 who presented with an injury. Triage narratives were interrogated using keywords to identify cases and describe visit characteristics. The relationship between: drug type, demographics (age, sex, ethnicity), triage acuity level, and year were examined. 84.4% of methamphetamine-related visits had a high triage acuity score. Compared to other visits methamphetamine-related visit more frequently required police or ambulance involvement. |
| Lee, SH. 2019.[44] | USA | Case identification | To compare machine learning algorithm’s ability to assign a syndromic category using chief complaint data only. | This retrospective validation study compared the performance of different machine learning models for disease surveillance by predicting a coded chief complaint using triage narratives. This study used chief complaint and discharge diagnosis narratives and compared them to coded diagnosis codes. The authors found that different machine learning models were better at predicting certain syndrome using different data sources and suggest that deep learning machine learning models may improve automatic coding of unstructured narrative data. |
| Marx, GE. 2019.[83] | USA | Case identification | To compare the performance of syndromic classifiers and physician review in identifying marijuana-associated ED visits using medical records. | This retrospective, observational validation cohort study assessed the validity of using syndromic surveillance data to detect marijuana-associated ED visits. The authors compared syndromic queries to physician-reviewed medical records in identifying marijuana-related visits. Keyword searches of triage narrative were compared to diagnostic code identified cases. Keyword identified cases, and diagnostic code + keyword identified cases were compared to expert identified "true cases". Descriptive statistics were used to report on patient demographics (age, sex, residential address), route of marijuana use, toxicology testing results, and if there was documented polysubstance use. For each visit, marijuana-specific diagnostic codes and keywords noted in the chief complaint or triage narrative were recorded. 453 of 44,942 total ED visits were identified as marijuana related; 188 (45%) of identified cases were true cases. All true positive cases were identified as marijuana-related using either diagnostic codes or triage keywords. PPV of each method varied by hospital (36% to 64%). 109 (58%) of true positive cases were men and 178 (95%) of cases used marijuana intentionally, visits were more likely to report versus smoked marijuana. |
| Nanda, G. 2019.[113] | AUS | Case identification | To compare different machine learning methods for identifying medical records that will require human review and assignment of rare cause-of-injury codes. | This two-stage retrospective descriptive, prospective cohort study compared the ability of different machine learning models to identify cases needing human review while assigning injury codes. The authors used four machine learning models to examine the triage narrative and determined that each model was able to detect rare cases needing human review. |
| Sterling, NW. 2019.[123] | USA | Predict outcomes | To examine machine learning algorithm’s ability to predict patient disposition using triage narrative alone. | This retrospective cohort study examined the ability of machine learning models to predict ED patient disposition. Using descriptive statistics and by comparing AUC the authors described the performance of three models (bag-of-words, paragraph vectors, and topic distributions) in predicting whether patients were discharged home, admitted to hospital, or left prior to completion of treatment. The mean character count for triage narratives was 143.17 (SD=77.8) and contained 64.3 words (SD=35.2). AUC of models were Bag-of-words= 0.737 (95% CI 0.734 - 0.740), Paragraph Vectors=0.785 (95% CI 0.782 - 0.788), Topic distribution=0.687 (95% CI 0.684 - 0.690). |
| Trivedi, TK. 2019.[93] | USA | Case identification | To identify injuries related to electric scooters and characterize their presentation patters and clinical outcomes. | This retrospective cohort study used keyword searches of triage narratives to identify patients presenting with injuries associated with electric scooter use. Flagged cases were reviewed, patient and visit characteristics were described. 58.2% of visits were male, the mean [SD] age was 33.7 [15.3] years. Injuries were described and the majority of patients ([94.0%) were discharged and helmet use rates were noted to be low. |
| Xingyu Zhang, M. 2019.[130] | USA | Predict outcomes | To describe the effects of socioeconomic, demographic and clinical factors on diagnostic imaging use and to develop a machine learning algorithm that can predict diagnostic imaging in pediatric ED patients. | This study was a two-stage retrospective-descriptive and validation study that examined the association between ED visits and diagnostic imaging use in pediatric patients. The authors used machine learning to compare models that used: structured (vital signs), unstructured (free-text triage narratives), or a combination of structure and unstructured data. There were 27,665 visits included, 8394 (30.3%) visits received diagnostic imaging - 6922 (25.0%) visits received an X-ray and 1367 (4.9%) computed tomography scan. The model using structured variables had a c-statistic of C=0.71 (95% CI 0.70–0.71) for any imaging use, C=0.69 (95% CI 0.68–0.70) for X-ray, and C=0.77 (95% CI 0.76–0.78) for computed tomography. machine learning models including only unstructured information had higher predictive power C=0.81 (95% CI 0.81–0.82) for any imaging use, C=0.82 (95% CI 0.82-0.83) for X-ray, and C=0.85 (95% 95% CI 0.83–0.86) for computed tomography. Including both structured and unstructured data improved predictive power C=0.82 (95% 95% CI 0.82–0.83) for any imaging use, C=0.83 (95% 95% CI 0.83–0.84) for X-ray, and C=0.87 (95% 95% CI 0.86–0.88) for computed tomography. |
| Zhang, X. 2019.[129] | USA | Predict outcomes | To predict advanced diagnostic imaging use the data immediately available during ED triage. | This retrospective cohort study was a secondary analysis that examined the association between advanced diagnostic imaging utilization and the structured and unstructured information available during ED triage of adult patients. The authors compared models using structured, unstructured (triage narrative), and structured plus unstructured data. Structured data included demographics (age, sex, ethnicity), residence (private residence, nursing home, homeless, or other), insurance status, conveyance (ambulance or not), visit characteristics (arrival time, previous 72hr visit, initial versus follow-up visit), vital signs (pain scale, temperature, heart rate, respiratory rate, blood pressure, pulse oximetry), chief complaint codes, medical history, and triage acuity score. Unstructured data (reason for visit triage narrative) were extracted using natural language processing. There were 139,150 visits included, 21.9% resulted in advanced diagnostic imaging utilization: 16.8% who received computed tomography, 3.6% who received an ultrasound, 0.4% who received magnetic resonance imaging, and 1.2% of received multiple diagnostic imaging types. The AUC ranged from 0.69 to 0.83 depending on the diagnostic imaging exam assessed and the variables included in the predictive model: the inclusion of unstructured data improved the accuracy of all models. |
| Bacchi, S. 2020.[119] | AUS | Predict outcomes | To compare different machine learning models discriminatory power in predicting length of stay and discharge destination. | This retrospective, observational validation cohort study examined whether length of stay and discharge destination could be predicted using natural language processing and machine learning models. The authors examined the discriminatory power predicting whether patients would have admissions (≤2 days or>2 days) using the grouped unstructured data derived from nursing triage and physician admission notes. The artificial neural network model had the highest predictive power of r admissions >2days (AUC=0.75). For the prediction of home as a discharge destination all machine learning models performed similarly. |
| Fernandes, M. 2020.[127] | PRT | Predict outcomes | To describe a machine learning algorithms ability to predict the risk of mortality for patients at triage. | This retrospective cohort study evaluated a machine learning models’ ability to predict which patients are at risk of requiring cardiopulmonary resuscitation or dying within 24 hours from triage. machine learning models using structured data only were compared to those that included unstructured data. Structured data included vital signs (temperature, heart and respiratory rate, blood pressure, pulse oximetry, Glasgow Coma Scale, blood glucose level, and pain scale), coded chief complaints, triage acuity level, Demographics (age, sex), arrival information (ambulance vs. walk-in, stretcher vs. wheelchair or ambulatory, time of triage) number of prior ED visits, and the number and type of exams performed at triage. Unstructured data was the triage narrative for chief complaint. Of the 235826 patients examined 1121 required resuscitation or died. The predictive power of models was: logistic regression AUC=0.93, random forests AUC=0.95, and extreme gradient boosting AUC=0.96. The predictor variables with highest importance were Glasgow coma score, patient age, pulse oximetry and arrival mode. The models using structured and unstructured data had higher recall most accurately identified patients at risk of death or requiring resuscitation. |
| Fernandes, M. 2020.[132] | PRT, USA | Predict outcomes | To describe a machine learning algorithms ability to identify patients with a high risk of intensive care unit admission. | This multinational retrospective cohort study examined the ability of a machine learning model to predict patients at high risk for intensive care admission from EDs in Portugal and the United States. machine learning models used different sets of variables: triage acuity, triage acuity + Clinical variables, triage acuity + clinical variables + chief complaint, and clinical variables + chief complaint. For both hospitals, the logistic regression model had the best performance, with AUC of 0.91 (95% CI 0.90-0.92) for the United States hospital and 0.85 (95% CI 0.83-0.86) for the Portuguese hospital. Vital sign derangement (Heart rate, pulse oximetry, respiratory rate and blood pressure) were the biggest predictors of ICU admission. machine learning models using clinical variables and the chief complaint presented had improved recall for patients who are at risk for ICU admission. |
| Jones, PG. 2020.[61] | NZL | Quality improvement | To determine the accuracy of coded chief complaint coding using triage narratives. | This retrospective cohort study examined the accuracy of chief complaint coding using expert re-triage of visits. Triage narratives were used by experts to assess the accuracy of assigned chief complaints by comparing actual and assigned chief complaints. Results were compared for concordance, by sex, age, ethnicity, and by presenting complaints. 852 of the 1,000 visits sampled were eligible, coded chief complaints agreed with clinical notes in 514 (60.3%) of cases. Overall agreement occurred in 732 (85.9%) cases. Patient age, sex and ethnicity did not influence coding accuracy. |
| Joseph, JW. 2020.[124] | USA | Predict outcomes | To compare the ability of machine learning models to ESI or vital sign triggers to predict critical illness using triage data. | This retrospective cross-sectional and prospective comparison study compared the predictive power of different machine learning models in identifying critically ill patients. The models used data collected at triage: vital signs, triage acuity scores (ESI < 3), and free-text chief complaints as inputs to identify patients who were admitted to the ICU or died within 24hours. 13.7% of patients were critically ill; the AUC for the simplest predictive models (vital sign and triage acuity) were 0.521 and 0.672 (95% CI 0.519-0.522 and 0.671-0.674), respectively. For machine learning models AUCs were 0.803 (95% CI 0.802–0.804) for logistic regression, 0.820 (95% CI 0.818–0.821) for gradient boosting, 0.811 (95% CI 0.807–0.815) for structured data neural network, and 0.851 (95% CI 0.849–0.852) for neural network models using both structure and textual data. |
| Klang, E. 2020.[56] | ISR | Predict outcomes | To develop a machine learning model that predicts a patients need for non-contrast head CT. | This retrospective cohort study developed a machine learning model to predict the need for non-contrast head computed tomography during triage. The models compared used coded and free text chief complaints derived from the triage narrative. Coded data was limited to triage specific data: demographics (age and sex), presentation data (admission date/time); conveyance (either walk-in, ambulance), number of previous visits, triage acuity score, and coded chief complaint. Free text complaints were derived from a two-word complaint recorded by the triage nurse. Of the included 595,561 visits computed tomography rate was 11.8%. Of each unique variable chief complaint had the AUC (0.87). The best model showed an AUC of 0.93 (95% CI 0.931–0.936) for predicting non-contrast head CT usage at triage level. |
| Klug, M. 2020.[57] | ISR | Predict outcomes | To evaluate a machine learning model for predicting mortality and triage level. | This retrospective cohort study trained and evaluated the performance of a machine learning model for predicting mortality using triage data. The authors evaluated each of the following as individual predictor variables: demographics (age and sex), presentation data (admission date/time); conveyance (either walk-in, ambulance), number of previous visits, triage acuity score, home medications, comorbidities, and coded chief complaint. Early and short-term mortality data (within 2 and 30 days of triage, respectively) was derived from electronic health records and vital statistics registries. Of the 799,522 ED visits the early mortality rates were 0.6% and 2.5%, respectively. The machine learning Model utilizing the full data set had an AUC of 0.962 for early and 0.923 for short-term mortality. The machine learning model using the nine most predictive variables (age, arrival mode, chief complaint, five primary vital signs, and triage acuity) had an AUC of 0.962 for early mortality. |
| Mor, S. 2020.[58] | ISR | Quality improvement | To describe how frequently family history of coronary artery disease is collected at triage and to compare the clinical characteristic of patients with and without family history. | This retrospective cohort describes how frequently clinicians recorded family history of coronary artery disease and whether it is predictive of ST elevation myocardial infarction. The following clinical risk factor information was collected: demographics (age, sex, and ethnicity), family history of coronary artery disease, and medical history (diabetes, dyslipidemia, hypertension, previous cardiac events, and smoking). Outcome variables examined included triage acuity score assignment, time to: nursing care, ECG, physician assessment and diagnosis, hospital length of stay, readmission rates, and mortality. Compared to physicians’ nurses collected family history less frequently (98.8% vs. 5.7%). |
| Stapelberg, NJC. 2020.[97] | AUS | Case identification | To validate a machine learning algorithm that can identify suicidal and self-harm presentations to the ED. | This retrospective cohort study used visit data from 2 EDs to validate a machine learning algorithm to identify suicidal ideation and self harm presentations. The psychiatrist evaluated dataset was used to determine factor weights and the resulting machine learning algorithm achieved a sensitivity of 0.95 and a specificity of 0.92 in identify cases. |
| Robinson, J. 2020.[98] | AUS | Case identification | To describe the machine learning language processing models and data elements that were used to develop a self-harm surveillance system. | This retrospective cohort study details the development of a regional self-harm surveillance system. Cases will be identified using diagnostic and billing codes and free text triage notes. A machine learning model will identify ED visits for self harm using data collected during ED visits: demographics (age, sex, residential address), visit characteristics (date and time), clinical characteristics (categorical chief complaint, free-text triage narratives, diagnostic codes), and treatment information (time to assessment, disposition). These fields will be used to identify cases, mechanism of injury, and medication involvement data. Mechanisms of injury will be grouped as: Injuries (intentional injury, use of firearms or explosives, sharp objects, falls from a height, and other); drug related (medication or drug, alcohol, poison/chemical/noxious substances); and hanging, strangulation, suffocation, drowning, or submersion. |
| Roquette, BP. 2020.[120] | BRA | Predict outcomes | To compare different machine learning models’ ability to predict admission using both structured and unstructured triage data. | This retrospective observational study evaluated a machine learning models predictive power for patients requiring hospital admission using both structured and unstructured data available at triage for patients under 18 years of age. machine learning model were trained with structured clinical variables and unstructured (textual) clinical narratives. Structured variables included: Demographics (age, sex, city of birth,), registration details (insurance statues, number and time from previous ED visits, home residence), historical variables (number of past image exam requests, number of past laboratory test requests, number of past ED visits, number of prior admissions, past image exam/visit ratio, past admission/visit ratio, past laboratory exam/visit ratio), clinical variables (triage acuity score, categorical symptom,); time variables (arrival time, time to triage); vital signs (heart rate, blood pressure, oxygen saturation, temperature, weight, blood glucose, pain score); and unstructured narrative data that included (drugs, text triage notes, previous triage notes, chief complaint, past visit chief complaint, previous visit medical image exam requests). There was a 5.76% admission rate in the studied period. The machine learning model achieved an AUC of 0.892. |
| Sterling, NW. 2020.[126] | USA | Predict outcomes | To compare the predictive power of machine learning models to human raters in predicting triage acuity using triage narratives. | This retrospective cohort and prospective model validation study trained and validated a machine learning model to predict ED resources requirements using triage acuity as a proxy. Using natural language processing Spielberg of triage narratives, the researchers compared the predictive power of machine learning models to human experts using structured (demographics, age, sex, vital signs, previous visit information, arrival mode, coded chief complaint, and coded medical history variables and unstructured triage narratives. The machine learning model accuracy was comparable to expert raters (65.9% vs 69.0%). machine learning models more accurately predicted low resource visits. |
| Sveticic, J. 2020.[100] | AUS | Case identification | To compare the sensitivity and specificity of discharge codes in identifying suicide and self harm cases in a dataset that used machine learning of triage narratives to identify cases. | This retrospective cohort study evaluated the reliability of different methods for identifying suicide and self injury ED presentations. Charts identified by a machine learning algorithm as related to suicide were reviewed by an expert clinician and classified as dealing with: a suicide attempt, suicidal ideation, or non-suicidal self-injury. The diagnosis codes, presenting complaints, and patient/visit characteristics were compared. There was significant diagnostic and presenting complaints code heterogeneity. Diagnostic codes had low sensitivity in identifying suicide attempts (18.7%), non-suicide self-injury (38.5%) and suicidal ideation (42.3%) and were biased toward identifying cases associated with female gender, indigenous status, and cases involving knife related presentations. |
| Vernon, N. 2020.[41] | USA | Case identification | To describe the patterns and characteristics of patients presenting with injuries related to e-scooters. | This retrospective review used triage narratives to identify keywords associated with electric scooter injury ED visits. The authors used keyword searches of triage narratives to identify cases and describe the patterns and characteristics of patients presenting with injuries related to e-scooters. 293 patients were included. The mean patient age was 34.1 (SD=14.0), 56% of patients were male. 100% of patient received at least one diagnostic imaging test (number of tests ranged from 1-9 (mean=2.4). 276 (94.2%) patients were discharged from the ED, 5 (1.7%) were transferred, 1%, 2 (0.7%) were admitted. |
| Bouchouar, E. 2021.[74] | CAN | Case identification | To describe the process of developing and implementing a syndromic surveillance system. | This two stage study reports on the retrospective design and prospective validation of a syndromic surveillance system. The five stages include: the initial review of ED data; the development of syndrome definitions; The natural language processing model development; validation of machine learning model; and refinement of the machine learning model. ED data examined included: coded chief complaints, triage narratives, discharge diagnosis codes. Validation and refinement measured the positive predictive value (the total number of true-positive over the total number of records). Each data fields influence on syndromic assignment was measured individually and in combination. The narrative terms were common in each syndrome were measured as frequencies. The positive predictive value for the automated detection of each syndrome ranged from 48.8–89.5% to 62.5–94.1%. |
| Cheung, KY. 2021.[62] | CHN | Quality improvement | To examine the validity of triage by comparing triage nurse assigned acuity scores with expert review and rates of resuscitation in older adults. | This retrospective descriptive, cross sectional study re-examined triage acuity scores by: comparing assigned categories and scores against an expert panel and by examining for whether patients required life-saving interventions in adults greater than 65 years of age. Using the clinical narrative and structured variables (assigned complaint category and vital signs) experts re-triaged patients. Sensitivity, specificity, and predictive values were used to summarise whether patients who received resuscitation were correctly assigned in to the highest triage scores. Percentage agreement and weighted kappa were used assess agreement on triage acuity levels and rates of: correct, over, and under-triage. Overall expert panel agreement was 96.7%. The agreement between clinical triage category and the expert panel assignment was 68.5%; with 16.3% and 15.3% over-triage and under-triage rates, respectively. Weighting kappa between groups was 0.72 (95% CI 0.53–0.91). The sensitivity, specificity and positive likelihood ratio for the need for life-saving interventions were 75.0% (95% CI 47.6%–92.7%), 97.1% (95% CI 94.4%–98.8%), and 26.2 (95% CI 12.5%–54.8%), respectively. |
| Delany, C. 2021.[84] | AUS | Case identification | To describe the effects of the 2018 Commonwealth Games on the volumes and distributions of drug and alcohol related ED presentations. | This retrospective observational study examined the impact of the 2018 Commonwealth Games on drug and alcohol related ED presentations. Using physician assigned diagnostic codes, and drug and alcohol related keyword searches of nurse recorded free-text triage narratives researchers identified cases. Presentation rates were compared to the same time period in the preceding year. Patient demographics (sex, country of residence,), presentation characteristics (arrival date/time, mode of arrival, triage acuity), and outcomes (discharge date/time, departure destination) were compared. There were 890 (5% of total) drug and alcohol-related ED presentations in the study period with no difference between pre (n = 312), during (n = 301) and post (n = 277) periods (P =0.2). Presentations were more common in younger (median age=35 years, IQR=24–48), male (n = 493,55%), Australian (n = 820, 92%) patients. most patients arrived by ambulance (n = 650, 73%), during the hours of 3PM and 11PM (n = 365, 41%). keyword searching identified 500 additional cases, the most commonly used terms were: ‘ETOH’ (n = 274, 55%), ‘overdose’/ ‘OD’ (n = 159, 32%), ‘intox’ (n =63,13%), ‘drink’ (n = 58, 12%) and ‘drug’ (n= 45, 9%). |
| Klang, E. 2021.[128] | USA | Predict outcomes | To develop a machine learning model to predict mortality during triage. | This retrospective study developed and compared different machine learning models to predict mortality using structured free-text narrative notes. The study outcome was in-hospital death within 48 hours; structured and free-text data collected within 30 minutes from triage were included in the model. Variables include demographics (age, sex, and ethnicity); arrival mode (walk-in, by ambulance); clinical parameters (vital signs, triage acuity scores, coded chief complaints, laboratory orders); and nursing and physician free text information. The 48-hour mortality rate was 0.2%. the machine learning model AUC values ranged from AUC=0.97 (95% CI 0.96–0.99) to AUC=0.98 (95% CI 0.98–0.99), when combined the models AUC value was 0.99 (95% CI 0.98–0.99). |
| Ivanov, O. 2021.[125] | USA | Predict outcomes | To determine if different machine learning models could accurately assign triage scores. | This retrospective cohort study sought to determine if different machine learning models could accurately assign triage acuity scores. Using visit characteristics, free text narratives from triage, and patient history data machine learning model assigned triage acuity scores and original triage records were compared to expert clinician re-triage. Triage scores, mis-triage rates and AUC for triage accuracy were compared. machine learning model assigned acuity scores were more frequently accurate than the original nurse assigned score (75.7% vs. 59.8%, respectively p<0.001) and had better discernment on patients on the boundary between level 2 and 3 acuity assignments (80% vs. 41.4%, P <.001). |
| Klang, E. 2021.[133] | USA | Predict outcomes | To describe different machine learning models’ ability to predict neurovascular intensive care unit admission using data available in the first 30min of a visit. | This retrospective cohort study used structured and unstructured data available in the first 30min of an ED visit to predict Neuro-intensive care unit admission. The authors compared structured, unstructured and combined powers of machine learning models. Structured data included: demographics (age, sex, ethnicity, home address); visit characteristics (date/time, means of arrival, number of previous visits/admissions); clinical variables (coded chief complaint, triage vital signs, triage acuity score, and medical history codes); and free text (triage, nursing, and physician texts generated within the first 30minutes). 1900 (0.5%) of the 412,858 visits were admitted into Neuro-intensive care. The median daily number of ED presentations was 231 (IQR 200–256), the median time from triage to admission was 169 min (IQR 80–324). The machine learning models AUC values were 0.90 (95% CI 0.87–0.91), 0.92 (95% CI 0.91–0.94), and 0.93 (95% CI 0.92–0.95) for models trained with unstructured text data only, structured data-only, and the combined data model, respectively. |
| Lam, T. 2021.[85] | AUS | Case identification | To compare the overdose patterns and distributions for different pharmaceutical opioids. | This retrospective observational study examined ED records to measure and compare pharmaceutical opioids contribution to non-fatal overdoses. Researchers compared the volume of pharmaceutical opioids dispensed to ED records. ED cases were identified using diagnostic coding and keyword searches of triage narratives using of variations of generic and commercial opioid names. Records were reviewed and the following variables were extracted: patient demographics (age, sex, country of birth, home address); ED characteristics (location, type); clinical variables (triage acuity score, admission status, diagnostic codes); and overdose intent (accidental vs. intentional) and were compared by opioid types which were adjusted by annual supply rates - per 100 000 oral morphine equivalents (OME). The highest supply-adjusted overdose rates were seen with codeine (OME = 0.078, CI = 0.073–0.08) and oxycodone (OME =0.029, CI = 0.027–0.030); the lowest with tapentadol (OME = 0.004, CI = 0.003–0.006) and fentanyl (OME = 0.003, CI = 0.002–0.004). 62% of poisonings involved females. Codeine, oxycodone and tramadol overdose were associated with younger patients (59.5%, 41.7% and 49.8%, respectively) and intentional self-harm (65.2%, 50.6%, and 52.8%, respectively). |
| Metzger, P. 2021.[66] | USA | Quality improvement | To describe the impact of race and language on the triage acuity scores of patients controlled for illness severity. | This retrospective cohort study examined the impact race and language had on triage acuity scores in illness severity adjusted presentations in immunocompromised patients aged 0 to 17 years. Cases were identified by performing keyword searches of triage narratives for the following words: 'central line', 'diabetes' or 'diabetic ketoacidosis', 'hematology', 'neonatal', 'oncology', 'short gut', 'shunt', 'sickle cell', 'transplant' or 'bone marrow transplant'. The following variables were extracted: demographic information (age, sex, insurance status, race/ethnicity, language), clinical variables (vital signs, triage acuity level, coded chief complaint), and treatment variables (time to provider, LOS, disposition and National ED Over-Crowding Study score) and compared by ethnic status (white race vs non-white). There were 10,815 visits from 8,928 patients. White patients were 34.6% of patients. Non-White patients had reduced likelihoods of receiving an emergency (level 2) or urgent (level 3) acuity score (OR= 0.4, 95% CI 0.33–0.49; OR, 0.5, 95% CI 0.45–0.56, respectively) and greater likelihood of receiving a minor (level 5) acuity score. The disparity was not noted when comparing language independently of ethnicity. |
| Personnic, J. 2021.[112] | FRA | Case identification | To describe the frequency of neurological disorders in a pediatric ED. | This retrospective observational study described the frequency with which patients with neurological disorders presented to a pediatric ED under 17 years of age. This study used keyword searches of the nursing free-text narrative of the chief complaint recorded at triage. Age, sex, chief complaint, history of previous visit, neurology referral, types of investigations (bloodwork, lumbar puncture, and neuroimaging), and medical history data were collected. 1,471 charts with neurological chief complaints were identified (1.8% of total visits); of these patients, 806 (55%) received a final physician assigned neurologic diagnosis, 2% of whom were admitted into pediatric intensive care. Seizures were the most common diagnosis for admitted patients. 40% of patients had at least on ED visit in the preceding 6 months. |
| Rahilly-Tierney C. 2021.[86] | USA | Case identification | To create data linkages for patients with both ED and ambulance visits for opioid related overdoses. | This retrospective cohort studies compared the unstructured narratives from ambulance services to ED triage in order to make data linkages between the systems for patients who presented with opioid-related overdoses. There was an 82% and 63% match rate for ambulance and ED records, respectively, with a 3% false positive match rate between the systems. |
| Rodríguez Vico, A. 2021.[68] | ESP | Quality improvement | To evaluate the quality of triage and compare the number of signs and symptoms of acute stroke gathered at triage and by using commonly used stroke scales. | This retrospective cohort study compared the triage note complaints of confirmed acute strokes to other recorded stroke scales within the same patient chart. The most common presenting complaints were aphasia, ataxia, and limb weakness. The authors determined that simplified stroke assessment scales would detect fewer symptoms than more comprehensive tools. |
| Rozova, V. 2021.[99] | AUS | Case identification | To develop an automated system that can identify self-harm related ED presentations using triage narratives. | This retrospective cohort study evaluated triage notes for suicidal ideation or self-harm using the following methods using different natural language processing models and keyword searches. These were then classified by expert adjudicators who assigned whether the cases were self-harm, suicidal ideation, or false positives. natural language processing outperformed keyword searches. |
| Tahayori, B. 2021.[122] | AUS | Predict outcomes | To compare machine learning models to expert reviewers’ ability to predict ED disposition using triage narratives. | This retrospective cohort study used natural language processing of triage notes to predict ED disposition. The predictive power of the machine learning model was compared to expert consultant predictions. The machine learning model had an AUC of 0.88. The machine learning model had a predictive power that was close to expert consultants in general and was more sensitivity than expert review. |
