# Supplementary material for: The Use and Structure of Emergency Nurses’ Triage Narrative Data: Scoping Review
Source: JMIR Nurs. 2023 Jan 13;6:e41331. doi: 10.2196/41331 (PMC9883744; doi:10.2196/41331)
Supplement: Multimedia Appendix 2 [file nursing_v6i1e41331_app2.docx]

Multimedia appendix 2. Data items collected

| Study ID | Narrative | Age | Sex^i^ | Chief complaint code | Discharge status | Triage acuity score | Triage system | Arrival means | Arrival day | Arrival time | Vital signs | Diagnosis | Admission location | Alcohol or drug use | Diagnostic imaging use | Mortality | Ethnicity^ii^ | Length of stay | Pain | Syndrome classification | Treatment details | Labs | Physician narrative | Insurance | Trauma |
| --- | --- | --- | --- | --- | --- | --- | --- | --- | --- | --- | --- | --- | --- | --- | --- | --- | --- | --- | --- | --- | --- | --- | --- | --- | --- |
| Kabir, A. 1998.[47] | **X** |  |  |  | **X** |  |  | **X** | **X** |  |  | **X** |  |  |  |  |  |  |  |  |  |  |  |  |  |
| Beveridge, R. 1999.[48] | **X** |  |  |  |  | **X** | **X** |  |  |  |  |  |  |  |  |  |  |  |  |  |  |  | **X** |  |  |
| Goodacre, SW. 1999.[49] | **X** |  |  |  | **X** |  | **X** |  |  |  |  |  |  |  |  |  |  |  |  |  |  |  |  |  |  |
| Aronsky, D. 2001.[42] | **X** |  |  |  | **X** |  |  |  |  |  |  |  |  |  |  |  |  |  |  |  |  |  |  |  |  |
| Burt, CW. 2001.[87] | **X** | **X** | **X** | **X** |  | **X** |  |  |  |  |  |  |  | **X** |  |  | **X** | **X** |  |  |  |  | **X** |  | **X** |
| Howe, A. 2002.[106] | **X** | **X** | **X** |  |  |  |  |  |  | **X** | **X** |  |  | **X** |  |  |  |  |  |  |  |  |  |  |  |
| Begier, EM. 2003.[70] | **X** |  |  | **X** | **X** |  |  |  |  |  |  |  |  |  |  |  |  |  |  |  | **X** |  |  |  |  |
| Travers, DA. 2003.[75] | **X** |  |  |  | **X** |  |  |  |  |  |  | **X** |  |  |  |  |  |  |  |  | **X** |  |  |  |  |
| Chapman, WW. 2004.[104] | **X** |  |  | **X** |  |  |  |  |  |  |  |  |  |  |  |  |  |  |  |  | **X** |  |  | **X** |  |
| Day, FC. 2004.[43] | **X** |  |  | **X** | **X** |  |  |  |  |  |  |  |  |  |  |  |  |  |  |  |  |  |  |  |  |
| Mikosz CA. 2004.[134] | **X** |  |  |  | **X** |  |  |  |  |  |  |  |  |  |  |  |  |  |  |  |  |  |  |  |  |
| Baumann, MR. 2005.[60] | **X** | **X** | **X** |  | **X** | **X** | **X** | **X** |  |  |  | **X** | **X** |  | **X** |  | **X** | **X** | **X** |  |  | **X** | **X** |  |  |
| Chapman, WW. 2005.[71] | **X** |  |  | **X** |  |  |  |  |  |  |  |  |  |  |  |  |  |  |  |  | **X** |  |  |  |  |
| Chapman, WW. 2005.[72] | **X** |  |  | **X** | **X** |  |  |  |  |  |  |  |  |  |  |  |  |  |  |  |  |  |  |  |  |
| Muscatello, DJ. 2005.[73] | **X** | **X** | **X** | **X** |  | **X** | **X** |  | **X** |  |  |  |  |  | **X** |  |  |  |  |  |  |  |  |  |  |
| Thompson, DA. 2006.[38] | **X** | **X** | **X** | **X** | **X** | **X** |  |  | **X** |  |  |  |  |  |  |  |  | **X** |  |  | **X** |  |  |  |  |
| Gillam, C. 2007.[116] | **X** |  |  |  |  |  |  |  |  |  |  |  |  | **X** |  |  |  |  |  |  |  |  |  |  |  |
| Indig, D. 2008.[77] | **X** | **X** | **X** | **X** | **X** | **X** |  | **X** | **X** | **X** |  |  | **X** |  | **X** | **X** |  |  |  |  |  |  |  |  |  |
| Irvine, AK. 2008.[114] | **X** |  |  |  |  |  |  |  |  |  |  |  |  |  |  |  |  |  |  |  |  |  |  |  |  |
| Indig, D. 2009.[78] | **X** | **X** | **X** | **X** |  | **X** |  |  | **X** | **X** | **X** |  |  | **X** |  | **X** |  |  |  |  |  |  |  | **X** |  |
| Mitchell, R. 2009.[88] | **X** | **X** | **X** | **X** |  | **X** |  |  | **X** |  |  |  | **X** | **X** |  |  |  |  |  |  |  |  |  |  |  |
| Indig, D. 2010.[39] | **X** | **X** | **X** | **X** | **X** | **X** | **X** | **X** | **X** | **X** | **X** |  | **X** |  | **X** | **X** |  |  |  |  |  |  |  |  |  |
| McKenzie, K. 2010.[108] | **X** |  |  | **X** | **X** |  |  |  |  |  |  |  |  |  |  |  |  |  |  |  |  |  |  |  |  |
| Wagholikar, AS. 2011.[50] | **X** |  |  |  | **X** |  |  |  |  |  |  |  |  |  |  |  |  |  |  |  |  |  |  |  |  |
| Bregman, B. 2012.[109] | **X** | **X** | **X** |  |  |  |  |  |  | **X** | **X** |  |  |  |  |  |  |  |  |  |  |  |  |  |  |
| Grossmann, FF. 2012.[51] | **X** | **X** | **X** | **X** | **X** | **X** | **X** | **X** | **X** |  |  | **X** |  |  | **X** |  |  | **X** | **X** |  |  | **X** |  |  |  |
| Malmström, T. 2012.[52] | **X** |  |  |  | **X** |  |  |  |  |  |  |  |  |  |  |  |  |  |  |  |  |  |  |  |  |
| Rhea, S. 2012.[103] | **X** | **X** |  | **X** | **X** |  |  |  |  |  |  |  |  |  |  |  |  |  |  |  |  |  |  |  |  |
| Genes, N. 2013.[115] | **X** |  |  |  |  |  |  |  |  | **X** | **X** | **X** |  |  |  |  |  |  |  |  |  |  |  |  |  |
| Mosley, I. 2013.[63] | **X** | **X** | **X** | **X** | **X** | **X** | **X** | **X** | **X** |  |  |  | **X** |  |  |  |  | **X** |  |  |  | **X** |  |  |  |
| Vallmuur, K. 2013.[79] | **X** | **X** | **X** | **X** |  |  |  |  |  | **X** | **X** |  | **X** | **X** |  | **X** |  |  |  |  |  |  |  |  |  |
| Haas, SW. 2014.[53] | **X** |  |  |  | **X** | **X** |  |  |  |  |  | **X** |  |  |  |  |  |  |  |  | **X** |  |  |  |  |
| Liljeqvist, HTG. 2014.[94] | **X** |  |  | **X** | **X** |  |  |  |  |  |  |  |  |  |  |  |  |  |  |  | **X** |  |  |  |  |
| Rhea, SK. 2014.[45] | **X** | **X** | **X** | **X** | **X** |  |  |  |  |  |  |  |  |  |  |  |  |  |  |  |  |  |  |  |  |
| Handly, N. 2015.[121] | **X** | **X** | **X** |  | **X** | **X** | **X** | **X** |  | **X** | **X** |  |  |  |  |  |  |  |  |  |  |  |  |  |  |
| Hides, L. 2015.[80] | **X** | **X** | **X** |  |  |  |  | **X** |  |  |  |  |  | **X** |  | **X** |  |  |  |  |  |  |  |  |  |
| Mitchell, RJ. 2015.[91] | **X** | **X** | **X** |  |  | **X** | **X** | **X** | **X** |  |  |  |  | **X** |  |  |  | **X** |  |  |  |  |  |  |  |
| Gray, SE. 2016.[69] | **X** |  |  |  |  |  |  |  |  |  |  |  |  |  |  |  |  |  |  |  |  |  |  |  |  |
| Luther, M. 2016.[101] | **X** | **X** | **X** | **X** |  |  |  |  |  | **X** | **X** |  |  |  |  | **X** |  |  |  |  |  |  |  |  |  |
| Rahme, E. 2016.[95] | **X** | **X** | **X** | **X** |  | **X** | **X** |  |  |  |  |  | **X** |  | **X** |  |  | **X** |  |  |  |  |  |  |  |
| Whitlam, G. 2016.[81] | **X** | **X** | **X** | **X** |  | **X** |  |  | **X** | **X** | **X** |  |  | **X** |  |  |  |  |  |  | **X** |  |  |  |  |
| Berendsen Russell, S. 2017.[67] | **X** |  |  |  | **X** |  | **X** |  |  |  |  |  |  |  |  |  |  |  |  |  |  |  |  |  |  |
| DeYoung, K. 2017.[40] | **X** |  |  | **X** | **X** |  |  |  |  | **X** |  |  |  |  |  | **X** |  |  |  |  |  |  |  | **X** |  |
| Horng, S. 2017.[105] | **X** | **X** | **X** | **X** |  | **X** | **X** | **X** |  |  |  |  |  |  | **X** |  |  | **X** |  | **X** |  |  |  |  |  |
| Kuramoto-Crawford, SJ. 2017.[96] | **X** | **X** | **X** | **X** | **X** |  |  |  |  | **X** | **X** |  |  |  |  |  |  |  |  |  |  |  |  |  |  |
| Kondis, J. 2017.[107] | **X** | **X** |  | **X** |  | **X** |  |  |  |  |  |  |  | **X** |  |  | **X** |  |  |  |  | **X** |  | **X** |  |
| Harduar Morano, L. 2017.[102] | **X** |  |  | **X** | **X** |  |  |  |  |  |  |  |  |  |  |  |  |  |  |  |  |  |  |  |  |
| Zhang, X. 2017.[118] | **X** | **X** | **X** | **X** |  | **X** |  | **X** | **X** | **X** | **X** | **X** | **X** | **X** |  |  |  |  |  | **X** |  |  |  |  | **X** |
| Chu, KH. 2018.[110] | **X** | **X** | **X** | **X** |  |  |  |  |  |  |  |  |  |  |  |  |  |  |  |  |  |  |  |  |  |
| Gligorijevic, D. 2018.[117] | **X** |  |  |  |  |  | **X** | **X** |  |  |  |  |  |  |  |  |  |  |  |  |  |  |  |  |  |
| Goldman- Mellor, S. 2018.[131] | **X** | **X** | **X** | **X** | **X** | **X** |  |  |  |  |  |  |  |  | **X** |  |  |  |  |  | **X** |  |  |  |  |
| Hargrove, J. 2018.[92] | **X** | **X** | **X** | **X** |  | **X** |  |  | **X** |  |  |  |  | **X** |  |  |  |  |  |  |  |  |  |  | **X** |
| Hendin, A. 2018.[65] | **X** | **X** | **X** | **X** | **X** | **X** | **X** | **X** | **X** |  |  | **X** |  |  | **X** |  | **X** |  | **X** | **X** |  | **X** | **X** | **X** |  |
| Nagabhushan, M. 2018.[111] | **X** |  |  | **X** |  | **X** |  |  |  |  |  |  |  |  | **X** |  | **X** | **X** |  |  |  |  |  | **X** |  |
| Petruniak, L. 2018.[64] | **X** | **X** | **X** | **X** |  |  | **X** | **X** | **X** | **X** | **X** | **X** |  |  |  |  |  |  |  |  |  |  |  |  |  |
| Rice, BT. 2018.[76] | **X** | **X** | **X** |  | **X** |  |  |  |  |  |  |  |  |  |  |  |  |  |  |  |  |  |  |  |  |
| Dehgani Soufi, M. 2018.[54] | **X** |  |  |  |  |  | **X** | **X** |  |  |  | **X** |  |  |  |  |  |  |  |  |  |  |  |  |  |
| Chen, M. 2019.[89] | **X** | **X** | **X** | **X** |  | **X** |  |  | **X** |  |  |  |  | **X** |  |  |  |  | **X** |  |  | **X** |  |  |  |
| Choi, SW. 2019.[59] | **X** | **X** | **X** |  | **X** |  | **X** |  | **X** | **X** | **X** | **X** |  |  |  |  |  |  |  | **X** |  |  |  |  |  |
| Eley, R. 2019.[90] | **X** | **X** |  | **X** | **X** |  | **X** | **X** |  |  |  |  |  |  |  |  |  |  |  |  |  |  |  |  |  |
| Greenbaum, NR. 2019.[55] | **X** | **X** | **X** |  | **X** |  | **X** | **X** |  |  |  | **X** |  |  |  |  |  |  |  |  |  |  |  |  |  |
| Jones, R. 2019.[82] | **X** | **X** | **X** | **X** |  | **X** | **X** | **X** | **X** | **X** | **X** |  | **X** | **X** |  |  |  |  |  |  | **X** |  |  |  |  |
| Lee, SH. 2019.[44] | **X** | **X** | **X** | **X** | **X** | **X** |  |  |  | **X** | **X** |  |  |  |  |  |  |  |  |  |  |  |  |  |  |
| Marx, GE. 2019.[83] | **X** | **X** | **X** | **X** | **X** |  |  |  |  |  |  |  |  |  |  | **X** |  |  |  |  |  |  | **X** |  |  |
| Nanda, G. 2019.[113] | **X** |  |  |  | **X** |  |  |  |  |  |  |  |  | **X** |  |  |  |  |  |  |  |  |  |  |  |
| Sterling, NW. 2019.[123] | **X** |  |  |  |  | **X** | **X** |  |  |  |  |  |  |  |  |  |  |  |  |  |  |  |  |  |  |
| Trivedi, TK. 2019.[93] | **X** | **X** | **X** | **X** |  | **X** | **X** | **X** | **X** | **X** | **X** |  |  | **X** |  | **X** | **X** |  | **X** |  |  |  |  |  |  |
| Xingyu Zhang, M. 2019.[130] | **X** | **X** | **X** |  | **X** |  |  |  | **X** | **X** | **X** | **X** | **X** | **X** |  | **X** | **X** |  |  | **X** |  |  |  |  | **X** |
| Zhang, X. 2019.[129] | **X** | **X** | **X** |  | **X** |  | **X** | **X** | **X** | **X** | **X** | **X** | **X** |  |  | **X** | **X** |  |  | **X** |  |  |  |  | **X** |
| Bacchi, S. 2020.[119] | **X** |  |  |  |  | **X** |  |  |  |  |  |  |  |  | **X** |  | **X** |  | **X** |  |  | **X** | **X** | **X** |  |
| Fernandes, M. 2020.[127] | **X** | **X** | **X** | **X** | **X** | **X** | **X** | **X** | **X** | **X** | **X** | **X** |  |  |  |  | **X** |  |  | **X** |  |  | **X** |  |  |
| Fernandes, M. 2020.[132] | **X** | **X** | **X** |  | **X** | **X** | **X** | **X** | **X** |  |  | **X** |  |  | **X** |  | **X** |  |  | **X** |  |  |  |  |  |
| Jones, PG. 2020.[61] | **X** | **X** | **X** |  | **X** |  |  |  |  | **X** | **X** |  | **X** |  |  |  |  |  |  |  |  |  |  |  |  |
| Joseph, JW . 2020.[124] | **X** |  |  | **X** | **X** | **X** | **X** | **X** |  |  |  | **X** |  |  | **X** |  |  | **X** |  |  |  |  |  |  |  |
| Klang, E. 2020.[56] | **X** | **X** | **X** | **X** |  | **X** | **X** | **X** |  |  | **X** | **X** |  |  |  | **X** |  |  |  |  |  |  |  |  |  |
| Klug, M. 2020.[57] | **X** | **X** | **X** |  | **X** |  | **X** | **X** | **X** | **X** |  | **X** |  |  |  |  |  | **X** |  |  |  |  |  |  |  |
| Mor, S. 2020.[58] | **X** | **X** | **X** | **X** |  |  | **X** | **X** |  |  |  |  | **X** |  | **X** |  |  | **X** | **X** |  |  | **X** |  |  |  |
| Stapelberg, NJC. 2020.[97] | **X** | **X** | **X** | **X** | **X** | **X** | **X** | **X** |  | **X** | **X** |  | **X** |  |  |  |  |  |  |  |  |  |  |  |  |
| Robinson, J. 2020.[98] | **X** | **X** | **X** | **X** | **X** | **X** |  |  |  | **X** | **X** |  | **X** | **X** |  |  |  |  | **X** |  |  |  |  |  |  |
| Roquette, BP. 2020.[120] | **X** | **X** | **X** |  | **X** |  | **X** | **X** |  |  |  | **X** |  |  |  |  |  |  | **X** | **X** |  |  |  |  | **X** |
| Sterling, NW. 2020.[126] | **X** | **X** | **X** |  |  |  | **X** |  |  |  |  | **X** | **X** |  |  |  | **X** |  |  | **X** |  | **X** | **X** |  |  |
| Sveticic, J. 2020.[100] | **X** | **X** | **X** | **X** | **X** |  | **X** |  |  |  |  |  | **X** | **X** |  |  |  |  |  |  |  | **X** |  |  |  |
| Vernon, N. 2020.[41] | **X** | **X** | **X** |  |  | **X** | **X** |  | **X** | **X** |  |  |  |  |  |  | **X** |  | **X** |  |  |  |  |  |  |
| Bouchouar, E. 2021.[74] | **X** |  |  | **X** | **X** |  | **X** |  |  |  |  |  |  |  |  |  |  |  |  |  |  |  |  |  |  |
| Cheung, KY. 2021.[62] | **X** | **X** | **X** |  |  | **X** |  | **X** | **X** |  |  |  |  |  |  |  |  |  |  |  |  |  |  |  |  |
| Delany, C. 2021.[84] | **X** | **X** | **X** | **X** |  | **X** | **X** | **X** | **X** | **X** | **X** |  | **X** |  |  | **X** |  |  | **X** |  |  |  |  |  |  |
| Klang, E. 2021.[128] | **X** | **X** | **X** | **X** | **X** | **X** | **X** | **X** | **X** |  |  | **X** | **X** |  |  |  |  | **X** |  |  |  |  | **X** | **X** |  |
| Ivanov, O. 2021.[125] | **X** | **X** | **X** |  | **X** |  | **X** | **X** | **X** | **X** | **X** | **X** |  |  |  | **X** |  |  |  | **X** |  |  |  |  |  |
| Klang, E. 2021.[133] | **X** | **X** | **X** | **X** | **X** | **X** | **X** | **X** | **X** |  |  | **X** | **X** |  | **X** |  |  | **X** |  |  |  |  |  | **X** |  |
| Lam, T. 2021.[85] | **X** | **X** | **X** | **X** |  | **X** | **X** | **X** |  |  |  |  | **X** |  |  | **X** |  |  |  |  |  |  |  |  |  |
| Metzger, P. 2021.[66] | **X** | **X** | **X** | **X** | **X** | **X** | **X** | **X** | **X** |  |  |  | **X** |  |  |  |  |  | **X** |  |  |  |  |  | **X** |
| Personnic, J. 2021.[112] | **X** | **X** | **X** | **X** | **X** |  |  |  |  |  |  |  |  |  | **X** |  | **X** |  |  |  |  |  | **X** |  |  |
| Rahilly-Tierney C. 2021.[86] | **X** | **X** | **X** | **X** | **X** |  |  |  | **X** | **X** | **X** | **X** |  |  |  | **X** |  |  |  |  |  |  |  |  |  |
| Rodríguez Vico, A. 2021.[68] | **X** | **X** | **X** | **X** |  |  |  |  |  |  |  |  |  |  |  |  |  |  |  |  |  |  |  |  |  |
| Rozova, V. 2021.[99] | **X** |  |  | **X** |  |  |  |  |  |  |  |  |  |  |  |  |  |  |  |  |  |  |  |  |  |
| Tahayori, B. 2021.[122] | **X** |  |  |  |  | **X** |  |  |  |  |  |  |  |  | **X** |  |  |  |  |  |  |  |  |  |  |
| Total | 96 | 63 | 60 | 57 | 53 | 43 | 41 | 35 | 32 | 29 | 26 | 25 | 22 | 19 | 17 | 16 | 14 | 14 | 12 | 11 | 10 | 10 | 10 | 9 | 7 |

i – Sex includes: sex and gender

ii – Ethnicity includes: Ethnicity, race, aboriginal status, and country of residence
